# Variability of mitochondrial ORFans hints at possible differences in the system of doubly uniparental inheritance of mitochondria among families of freshwater mussels (Bivalvia: Unionida)

**DOI:** 10.1186/s12862-019-1554-5

**Published:** 2019-12-19

**Authors:** Davide Guerra, Manuel Lopes-Lima, Elsa Froufe, Han Ming Gan, Paz Ondina, Rafaela Amaro, Michael W. Klunzinger, Claudia Callil, Vincent Prié, Arthur E. Bogan, Donald T. Stewart, Sophie Breton

**Affiliations:** 1 Département de Sciences Biologiques, Université de Montréal, Montréal, QC Canada; 2 CIBIO/InBIO - Research Center in Biodiversity and Genetic Resources, University of Porto, Campus Agrário de Vairão, Vairão, Portugal; 3 CIIMAR - Interdisciplinary Centre of Marine and Environmental Research, University of Porto, Matosinhos, Portugal; 4 Deakin Genomics Centre, School of Life and Environmental Sciences, Deakin University, Geelong, Victoria Australia; 5 Departamento de Zooloxía, Xenética e Antropoloxía Física, Facultade de Veterinaria, Universidade de Santiago de Compostela, Campus de Lugo, Lugo, Spain; 6 BWG Environmental, Brisbane, QLD Australia; 7 Mollusca, Department of Aquatic Zoology, Western Australian Museum, Welshpool, WA Australia; 8 School of Veterinary and Biological Sciences, Murdoch University, Perth, WA Australia; 9 ECOBiv - Ecology and Conservation of Bivalves Research Group, Department of Biology and Zoology, Federal University of Mato Grosso, Cuiabá, MT Brazil; 10 Institut Systématique Evolution Biodiversité ISYEB - Muséum National d’Histoire Naturelle, CNRS, Sorbonne Université, EPHE, Université des Antilles, Paris, France; 11 North Carolina Museum of Natural Sciences, Raleigh, NC USA; 12 Department of Biology, Acadia University, Wolfville, NS Canada

**Keywords:** Freshwater mussels, Doubly uniparental inheritance of mitochondrial DNA, mtDNA sequencing, Mitochondrial ORFan genes, Evolution of protein structures and functions, Mitochondria and sexual systems

## Abstract

**Background:**

Supernumerary ORFan genes (i.e., open reading frames without obvious homology to other genes) are present in the mitochondrial genomes of gonochoric freshwater mussels (Bivalvia: Unionida) showing doubly uniparental inheritance (DUI) of mitochondria. DUI is a system in which distinct female-transmitted and male-transmitted mitotypes coexist in a single species. In families Unionidae and Margaritiferidae, the transition from dioecy to hermaphroditism and the loss of DUI appear to be linked, and this event seems to affect the integrity of the ORFan genes. These observations led to the hypothesis that the ORFans have a role in DUI and/or sex determination. Complete mitochondrial genome sequences are however scarce for most families of freshwater mussels, therefore hindering a clear localization of DUI in the various lineages and a comprehensive understanding of the influence of the ORFans on DUI and sexual systems. Therefore, we sequenced and characterized eleven new mitogenomes from poorly sampled freshwater mussel families to gather information on the evolution and variability of the ORFan genes and their protein products.

**Results:**

We obtained ten complete plus one almost complete mitogenome sequence from ten representative species (gonochoric and hermaphroditic) of families Margaritiferidae, Hyriidae, Mulleriidae, and Iridinidae. ORFan genes are present only in DUI species from Margaritiferidae and Hyriidae, while non-DUI species from Hyriidae, Iridinidae, and Mulleriidae lack them completely, independently of their sexual system. Comparisons among the proteins translated from the newly characterized ORFans and already known ones provide evidence of conserved structures, as well as family-specific features.

**Conclusions:**

The ORFan proteins show a comparable organization of secondary structures among different families of freshwater mussels, which supports a conserved physiological role, but also have distinctive family-specific features. Given this latter observation and the fact that the ORFans can be either highly mutated or completely absent in species that secondarily lost DUI depending on their respective family, we hypothesize that some aspects of the connection among ORFans, sexual systems, and DUI may differ in the various lineages of unionids.

## Background

Many species of gonochoric bivalve molluscs from four different orders (Unionida, Mytilida, Venerida, Nuculanida) possess a peculiar mode of mitochondrial transmission called doubly uniparental inheritance, or DUI, which is particularly well-documented (~ 80 species) in freshwater mussels of the order Unionida [1, 2]. DUI basically consists of a cyclic parent-specific inheritance of two distinct mitochondrial (mt) genomes (or mtDNA), where females segregate only the so called F (“female-transmitted”) mtDNA in their eggs and males segregate only the other mitotype, named M (“male-transmitted”), in their sperm. The zygote is heteroplasmic but, depending on its sexual development in the subsequent stages, i.e. whether it will become a female or a male as an adult, an individual will transmit only one of these two types of mt genomes to the next generation [1]. DUI seems to be strictly associated with the gonochoristic sexual system of a particular species, as it was discovered that four species of unionids and one species of margaritiferid each appear to have lost independently their M mtDNA during the transition from dioecy to hermaphroditism [3]. Notice that, however, it is not known if the loss of the M type is perfectly contemporaneous with the switch to hermaphroditism. All these mentioned obligate hermaphroditic species now retain only a modified version of the F genome that is called the H genome (for “hermaphrodite”) [3]. In an attempt to understand if and how DUI and sex determination are connected, genomic studies have highlighted the following features of DUI in freshwater mussel mtDNAs: (1) a high level of sequence divergence between F and M (up to ~ 40% of difference in their nucleotide sequences, resulting into an almost 50% difference in the amino acid sequence of their encoded proteins [4, 5]); (2) the presence of a 3′-elongation of the *cox2* gene in the M mtDNA (compared to that found in most metazoans) [6]; (3) the presence in both mt genomes of “ORFan” genes, i.e., genes without obvious homology or function [7], named F-*orf* in the F mtDNA and M-*orf* in the M mtDNA, respectively [8]. An ORFan is found also in the H mtDNAs mentioned above: it is a highly mutated F-*orf* and is named H-*orf* for distinction [3]. It is worth noting that these features of DUI-positive freshwater mussel mtDNAs also appear in practically all DUI bivalves outside Unionida, although with many variations and combinations (e.g., F vs M divergence can be lower; elongated *cox2* genes can appear in the F instead of the M, or be absent from both; and mtDNA-specific ORFans can be present in only one of the two mtDNAs or duplicated in a same mt genome [1, 5, 9–12]). Additional coding sequences are sometimes found in the mtDNA of bivalves (with or without DUI) and even other molluscan species [9, 13–22]. Usually they are identifiable as duplicated standard mitochondrial protein coding genes, with a variable degree of similarity to the original sequence. In fact, the two copies can evolve in tandem but, in other cases, the only recognizable parts consist of small segments encoding functional domains of the original mitochondrial genes. In absence of further functional studies, this leads us to hypothesize that the primary function of these genes, if indeed they retain functionality, might be related to oxidative phosphorylation.

Functional studies focusing on the ORFan genes F-*orf* and M-*orf* in DUI bivalves provided evidence they are transcribed and translated into proteins (from here on respectively indicated as F-*ORF* and M-*ORF*) located inside and outside the mitochondria [3, 10, 23–26]. Moreover, extensive bioinformatic studies on both the gene sequences and their translated proteins produced evidence for two options for the origin of the ORFans, which may be the result of either (1) the insertion of viral sequences into the host mt genome [23, 27] or (2) the duplication and subsequent modification of extant mitochondrial genes and sequences [5, 28]. Analyses aimed at understanding their origin by predicting the functions of their protein products broadly converged on similar patterns in all species considered (freshwater mussels and others). F-*ORF*s and M-*ORF*s, for example, were predicted to interact with nucleic acids and/or membranes (for signaling and/or interactions with the immune system), and M-*ORF*s, in particular, were also predicted to interact with the cytoskeleton and to have a role in the ubiquitination processes [27, 28]. The high variability of the ORFans among distantly related families and orders, however, questions their homology in all DUI bivalves. To have a better understanding of ORFans evolution and the functions of their encoded proteins, efforts should focus on a taxon in which DUI is widespread, such as the Unionida [2]. Until a few years ago, only F, M, and H mt genomes from the family Unionidae and a few F and H genomes of Margaritiferidae were available, but recently mtDNAs from families Hyriidae, Iridinidae, Mulleriidae, and the first margaritiferid M mtDNAs have been published [5, 29]. Given the hypothesized, although still untested, link between ORFans and sexual systems in freshwater mussels [3], the sequencing of these new mt genomes allowed examination of the evolution of ORFan genes, DUI, and sexual systems in a phylogenetic context [5]. It was suggested that DUI may have been present as an ancestral state before the radiation of the order Unionida, and that some ORFans have been partially or totally purged from the remaining mtDNAs of some lineages that may have lost DUI in their early stages of radiation (Iridinidae and Mulleriidae). However, the ORFans have been maintained in the other families that regularly show DUI (Hyriidae, Margaritiferidae, Unionidae), and in each of these taxa, the mt genomes, especially the M, show their own family-specific peculiarities. For example, in margaritiferid M mtDNAs the M-*orf* is duplicated (one copy, M-*orf*1, appears to be homologous to the M-*orf* of Unionidae and Hyriidae, while the second copy, M-*orf*2, is specific to Margaritiferidae only), whereas the hyriid M-*orf* is much longer than those of Margaritiferidae and Unionidae [5].

In this study, we present eleven new mt genomes from ten species of freshwater mussels, with or without DUI and with different sexual systems (for each species, references describing DUI status and/or reproductive modes on which we relied for this study are given): *Chambardia rubens* (Lamarck, 1819) [30, 31] for Iridinidae; *Anodontites elongata* (Swainson, 1823) [32], *Fossula fossiculifera* (d’Orbigny, 1835) [33], *Lamproscapha ensiformis* (Spix and Wagner, 1827) (C. Callil personal observation), *Monocondylaea parchappii* (d’Orbigny, 1835) (C. Callil personal observation) for Mulleriidae; *Castalia ambigua* Lamarck, 1819 [34, 35], *Diplodon suavidicus* (Lea, 1856) [35], *Prisodon obliquus* Schumacher, 1817 [35], *Westralunio carteri* (Iredale, 1934) [36] for Hyriidae; and *Pseudunio auricularius* (Spengler, 1793) [37] for Margaritiferidae. First, we characterize the overall structure of the new mt genomes and highlight their unique features, some of which are described for the first time, and we build a phylogeny of freshwater mussels using these and other genomes. Then we identify new F-*orf* and M-*orf* genes and describe the new F-*ORF* and M-*ORF* proteins by comparing them to a set of already published sequences, showing how, despite having evolved different three-dimensional configurations, they share some key features. Finally, considering our findings, we discuss whether the DUI system works the same way in all DUI freshwater mussels or if there may be family-specific differences, as well as the modifications occurring in the mtDNAs after DUI is lost.

## Results

### Sequencing, assembly, and general features of the new mt genomes

We obtained a complete sequence for ten of the eleven new mt genomes; the M mtDNA of *W. carteri* showed a sequencing gap in a non-coding region between *trnV* and *trnH*. All sequences were deposited in GenBank under the accession numbers MK761136–46 (Table 1). A summary of their length and a comparison of their gene order are given in Table 2. In summary, they present the same general features recently highlighted by [5] (for families Iridinidae, Mulleriidae, Hyriidae, Margaritiferidae) and [29] (for Margaritiferidae). As is typical for DUI freshwater mussels, the *cox2* gene carried by *W. carteri* M mt genome is longer compared to its F counterpart (respectively 1329 bp and 693 bp) and to those of non-DUI species [6] (Table 3). Apart from the occasional species-specific difference in length of some non-coding regions, particularly in Hyriidae (i.e., between *atp8* and *nad4L* in *D. suavidicus*, and between *trnV* and *trnH* in *W. carteri* M mtDNA) and Iridinidae (1049 bp between *nad5* and *trnF* in *C. rubens*, compared to the 23-76 bp of the other species mtDNAs), the most notable features lie in the presence/absence of ORFans, on which we will focus.

**Table 1:** Summary of the newly sequenced species and their mt genomes

Family	Species	Country	Latitude	Longitude	Sexual system	GenBank accession numbers		
						non-DUI	F	M
Iridinidae	*Chambardia rubens*	Mauritania	16.33880	−11.97809	dioecious	MK761138		
Mulleriidae	*Anodontites elongata*	Brasil	−14.67936	−56.23611	dioecious	MK761136		
	*Fossula fossiculifera*	Brasil	−14.67936	−56.23611	dioecious	MK761140		
	*Lamproscapha ensiformis*	Brasil	−16.00573	−55.90867	unknown	MK761141		
	*Monocondylaea parchappii*	Brasil	−14.67936	−56.23611	hermaphroditic	MK761142		
Hyriidae	*Castalia ambigua*	Brasil	−14.67936	−56.23611	dioecious	MK761137		
	*Diplodon suavidicus*	Brasil	−6.018083	−60.194306	unknown	MK761139		
	*Prisodon obliquus*	Brasil	−6.018083	−60.194306	unknown	MK761143		
	*Westralunio carteri*	Australia	−33.30210	115.81770	dioecious		MK761145	MK761146
Margaritiferidae	*Pseudunio auricularius*	France	47.01710	0.56310	dioecious		MK761144	

For each species for which at least one mt genome sequence was obtained in this study (either complete or largely complete), the respective family, and the provenance of the specimen(s) used for the sequencing (columns ‘Country’, ‘Latitude’, ‘Longitude’) are indicated, as well as the respective sexual system. For each mtDNA sequenced, its type is specified: non-DUI., mtDNA of dioecious or hermaphroditic species with no evidence of DUI presence or secondary loss; F and M, female- and male-transmitted mtDNAs of dioecious DUI species, respectively. The only available mtDNA of *Pseudunio auricularius*, a dioecious species for which no evidence of DUI has been produced yet (no M mtDNA sequence available yet), which carries an F-*orf*, has been considered as F for simplicity

**Table 2:** Gene order comparison for the newly sequenced mt genomes

*Chambardia rubens*	*Anodontites elongatus*	*Fossula fossiculifera*	*Lamproscapha ensiformis*	*Monocondylaea parchappii*	*Pseudunio auricularius* F	*Castalia ambigua*	*Diplodon suavidicus*	*Prisodon obliquus*	*Westralunio carteri* F	*Westralunio carteri* M	
16,697 bp	15,255 bp	15,632 bp	15,583 bp	15,528 bp	16,195 bp	17,092 bp	18,211 bp	16,691 bp	16,766 bp	18,038 bp	
cox1	***cox1***	***cox1***	***cox1***	***cox1***	***cox1***	***cox1***	***cox1***	***cox1***	***cox1***	***cox1***	*
cox3	***cox3***	***cox3***	***cox3***	***cox3***	***cox3***	***cox3***	***cox3***	***cox3***	***cox3***	***cox3***	*
atp6	***atp6***	***atp6***	***atp6***	***atp6***	***atp6***	***atp6***	***atp6***	***atp6***	***atp6***	***atp6***	*
trnD	***trnD***	***trnD***	***trnD***	***trnD***	***trnD***	***trnD***	***trnD***	***trnD***	***trnD***	***atp8***	
atp8	***atp8***	***atp8***	***atp8***	***atp8***	***atp8***	***atp8***	***atp8***	***atp8***	***atp8***	***trnD***	
										**M-*orf***	
nad4L	***nad4L***	***nad4L***	***nad4L***	***nad4L***	***nad4L***	***nad4L***	***nad4L***	***nad4L***	***nad4L***	***nad4L***	*
nad4	***nad4***	***nad4***	***nad4***	***nad4***	***nad4***	***nad4***	***nad4***	***nad4***	***nad4***	***nad4***	*
*nad6*	*nad6*	*nad6*	*nad6*	*nad6*	*nad6*	*nad6*	*nad6*	*nad6*	*nad6*	*nad6*	*
*trnG*	*trnG*	*trnG*	*trnG*	*trnG*	*trnG*	*trnG*	*trnG*	*trnG*	*trnG*	*trnG*	*
										*trnQ*	
*nad1*	*nad1*	*nad1*	*nad1*	*nad1*	*nad1*	*nad1*	*nad1*	*nad1*	*nad1*	*nad1*	*
*trnL2*	*trnL2*	*trnL2*	*trnL2*	*trnL2*	*trnL2*	*trnL2*	*trnL2*	*trnL2*	*trnL2*	*trnL2*	*
*trnV*	*trnV*	*trnV*	*trnV*	*trnV*	*trnV*	*trnV*	*trnV*	*trnV*	*trnV*	*trnV*	*
*trnI*	*trnI*	*trnI*	*trnI*	*trnI*	*trnI*	*trnI*	*trnI*	*trnI*	*trnI*		
*trnC*	*trnC*	*trnC*	*trnC*	*trnC*	*trnC*	*trnQ*	*trnQ*	*trnQ*	*trnQ*		
*trnQ*	*trnQ*	*trnQ*	*trnQ*	*trnQ*	*trnQ*	*trnC*	*trnC*	*trnC*	*trnC*	***trnH***	
nad5	***nad5***	***nad5***	***nad5***	***nad5***	***nad5***	***nad5***	***nad5***	***nad5***	***nad5***	***nad5***	*
*trnF*	*trnF*	*trnF*	*trnF*	*trnF*	*trnF*	*trnF*	*trnF*	*trnF*	*trnF*	*trnF*	*
*cob*	*cob*	*cob*	*cob*	*cob*	*cob*	*cob*	*cob*	*cob*	*cob*	*cob*	*
*trnP*	*trnP*	*trnP*	*trnP*	*trnP*	*trnP*	*trnP*	*trnP*	*trnP*	*trnP*	*trnP*	*
					*trnE*						
*trnN*	*trnN*	*trnN*	*trnN*	*trnN*	*trnN*	*trnN*	*trnN*	*trnN*	*trnN*	*trnN*	*
*trnL1*	*trnL1*	*trnL1*	*trnL1*	*trnL1*	*trnL1*	*trnL1*	*trnL1*	*trnL1*	*trnL1*	*trnL1*	*
*rrnL*	*rrnL*	*rrnL*	*rrnL*	*rrnL*	*rrnL*	*rrnL*	*rrnL*	*rrnL*	*rrnL*	*rrnL*	*
*trnY*	*trnY*	*trnY*	*trnY*	*trnY*	*trnY*	*trnY*	*trnY*	*trnY*	*trnY*	*trnY*	*
*trnT*	*trnT*	*trnT*	*trnT*	*trnT*	*trnT*	*trnT*	*trnT*	*trnT*	*trnT*	*trnT*	*
*trnK*	*trnK*	*trnK*	*trnK*	*trnK*	*trnK*	*trnK*	*trnK*	*trnK*	*trnK*	*trnK*	*
*rrnS*	*rrnS*	*rrnS*	*rrnS*	*rrnS*	*rrnS*	*rrnS*	*rrnS*	*rrnS*	*rrnS*	*rrnS*	*
*trnR*	*trnR*	*trnR*	*trnR*	*trnR*	*trnR*	*trnR*	*trnR*	*trnR*	*trnR*	*trnR*	*
*trnW*	*trnW*	*trnW*	*trnW*	*trnW*	*trnW*	*trnW*	*trnW*	*trnW*	*trnW*	*trnW*	*
*trnM*	*trnM*	*trnM*	*trnM*	*trnM*	*trnM*	*trnM*	*trnM*	*trnM*	*trnM*	*trnM*	*
*nad2*	*nad2*	*nad2*	*nad2*	*nad2*	*nad2*	*nad2*	*nad2*	*nad2*	*nad2*	*nad2*	*
					F-*orf*				F-*orf*		
*trnE*	*trnE*	*trnE*	*trnE*	*trnE*		*trnE*	*trnE*	*trnE*	*trnE*	*trnE*	
*trnS1*	*trnS1*	*trnS1*	*trnS1*	*trnS1*	*trnS1*	*trnS1*	*trnS1*	*trnS1*	*trnS1*	*trnS1*	*
										*trnI*	
*trnS2*	*trnS2*	*trnS2*	*trnS2*	*trnS2*	*trnS2*	*trnS2*	*trnS2*	*trnS2*	*trnS2*	*trnS2*	*
*trnA*	*trnA*	*trnA*	*trnA*	*trnA*	*trnA*	*trnA*	*trnA*	*trnA*	*trnA*	*trnA*	*
trnH	***trnH***	***trnH***	***trnH***	***trnH***	***trnH***	***trnH***	***trnH***	***trnH***	***trnH***	*trnC*	
nad3	***nad3***	***nad3***	***nad3***	***nad3***	***nad3***	***nad3***	***nad3***	***nad3***	***nad3***	***nad3***	*
cox2	***cox2***	***cox2***	***cox2***	***cox2***	***cox2***	***cox2***	***cox2***	***cox2***	***cox2***	***cox2***	*

Under every species name is indicated the length of the mtDNA. Genes in bold are encoded in forward direction, while genes in normal font in reverse direction. Gaps between genes in the table are present only as a consequence of the alignment of identical gene blocks. Positions with the same gene in every mtDNA are indicated with an asterisk (*) at the right side of the table

**Table 3:** C*ox2* gene length variability in freshwater mussels

Family	mtDNA		Number of *cox2* analyzed	Length (bp)			
	transmitted by	total number		minimum	maximum	mean	SD
Iridinidae	eggs	2	2	681	681	681.0	0.0
Mulleriidae	eggs	5	4	681	681	681.0	0.0
Hyriidae	eggs	5	5	681	693	683.4	4.8
	sperm	2	2	1329	1380	1354.5	25.5
Margaritiferidae	eggs	7	7	600	693	672.4	29.9
	sperm	3	3	1215	1224	1218.0	4.2
Unionidae	eggs	21	21	681	684	681.1	0.6
	sperm	17	17	825	1272	1193.2	130.8

Range, mean, and standard deviation (SD) of the *cox2* gene length in egg- and sperm-transmitted mtDNAs in different freshwater mussel families. Sperm-transmitted mtDNAs are present only in families showing DUI. Uniparental transmission of mtDNA through female gametes is assumed in Iridinidae and Mulleriidae, which do not show DUI. In Mulleriidae, the partially sequenced *cox2* gene from *Anodontites trapesialis* was excluded from this analysis; however, it does not show the typical 3′ elongation of M *cox2* genes [5]

### Phylogeny of freshwater mussel mt genomes

A Bayesian inference analysis was performed with MrBayes [38] on 12 protein coding genes and their respective protein sequences extracted from the new 11 mt genomes, from 51 additional mtDNAs of freshwater mussels available in GenBank (25 F, 22 M, and other 15 mtDNAs from non-DUI species; Additional file 1: Table S1), and from three outgroup species (the bivalves *Neotrigonia margaritacea* and *Solemya velum*, plus a member of the Caudofoveata, *Chaetoderma nitidulum*). The evolutionary models calculated for the aligned and trimmed gene sequences were ‘GTR + G’ for *nad4L* and ‘GTR + I + G’ for all others. The most supported model for the trimmed protein alignment was ‘Jones’ (posterior probability = 1.000). The nucleotide- and amino acid-based phylogenetic reconstructions reached convergence (standard deviation of split frequencies stabilized at values < 0.01) respectively after 39,000 and 17,000 generations.

**Fig. 1:**
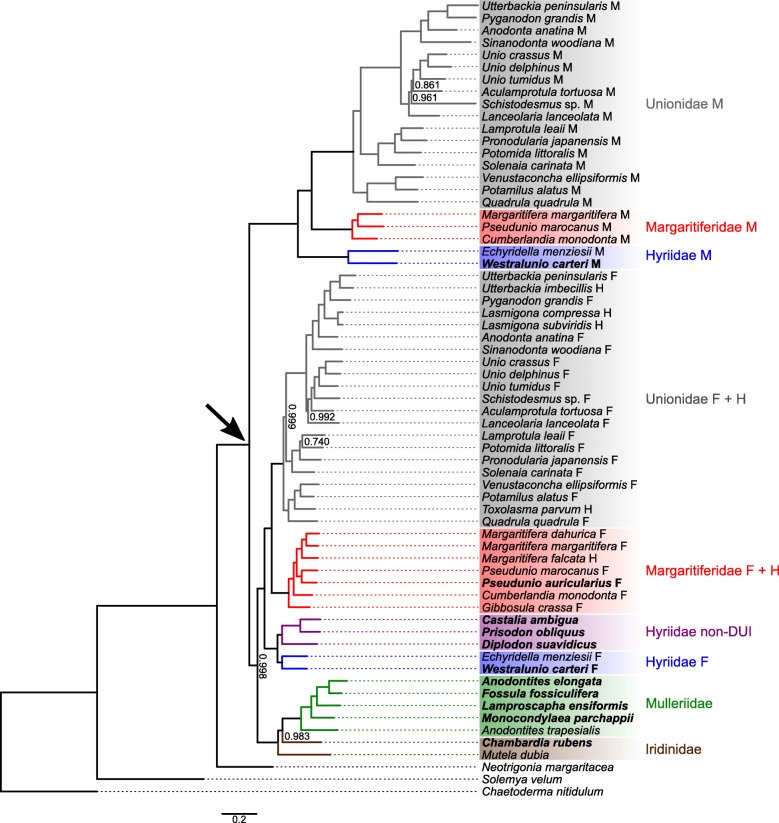
Bayesian inference phylogenetic tree of freshwater mussel mt genomes based on nucleotide sequences from 12 of their protein coding genes (*atp8* was excluded). All nodes have posterior probability 1.000, except where indicated. An arrow indicates the split of freshwater mussel M mtDNAs clade. Clades and groups of mtDNAs are color coded according to the family and/or type of mtDNA indicated on the right side of the figure. The new mtDNAs sequenced in this study are in bold character

In the nucleotide-based tree (Fig. 1), freshwater mussel mtDNAs form a monophyletic group divided in two main branches: one containing all M mtDNAs and another containing all female-transmitted ones, from both DUI and non-DUI taxa. Relationships among DUI species in these two main branches are maintained in most cases, i.e. the phylogeny of F mt genomes mirrors that of M ones. In the few cases where this situation does not occur, either the nodes usually have posterior probabilities < 1.000 (e.g., see the different relative position of *Aculamprotula tortuosa* mt genomes in the Unionidae clades) or the number of F and M genomes for a taxon is different (e.g., Margaritiferidae). In both of the two main branches, family Hyriidae is sister group to Margaritiferidae and Unionidae, which always form reciprocally sister groups. For Hyriidae clades, *W. carteri* mt genomes are always sister to the respective *Echyridella menziesii* ones. In Margaritiferidae, *P. auricularius* female-transmitted mtDNA is sister to *Pseudunio marocanus* F mtDNA. Iridinidae and Mulleriidae form a single branch sister to Hyriidae + Margaritiferidae + Unionidae female-transmitted mtDNAs, where Mulleriidae form a monophyletic clade but Iridinidae do not: *C. rubens* is sister to all Mulleriidae (node posterior probability = 0.983), while the other iridinid *Mutela dubia* is sister to all. Inside Mulleriidae, the dioecious [32] *A. elongata* is recovered as distantly related to the congeneric hermaphrodite [39–41] *Anodontites trapesialis*.

**Fig. 2:**
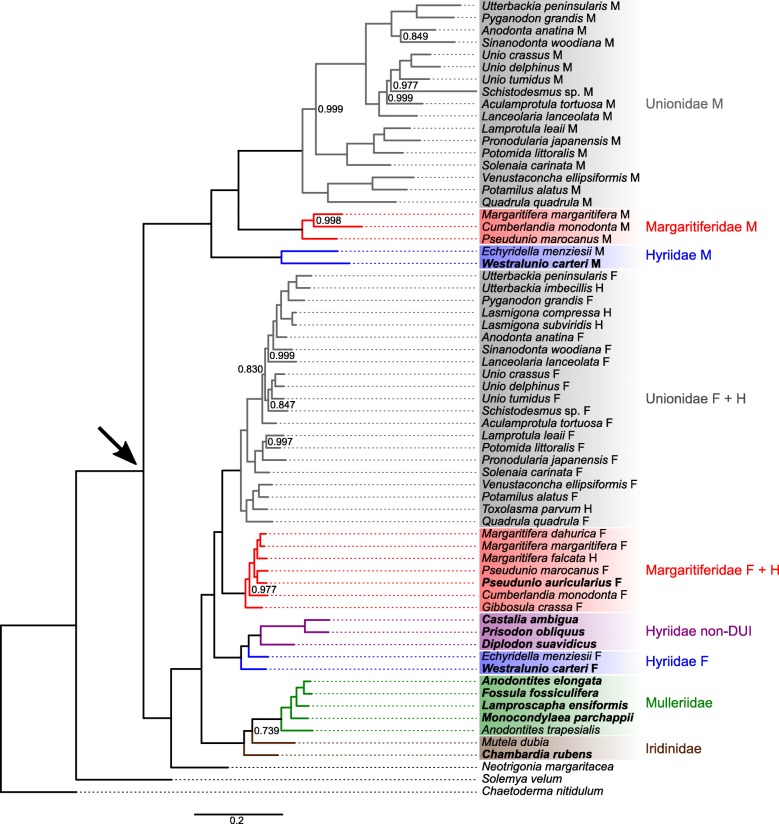
Bayesian inference phylogenetic tree of freshwater mussel mt genomes based on protein sequences translated from 12 of their protein coding genes (*atp8* was excluded). All nodes have posterior probability 1.000, except where indicated. An arrow indicates the split of freshwater mussel M mtDNAs clade. Clades and groups of mtDNAs are color coded according to the family and/or type of mtDNA indicated on the right side of the figure. The new mtDNAs sequenced in this study are in bold character

The main difference of the protein-based tree (Fig. 2) with the nucleotide-based one is the position of the M mtDNAs clade: here, it is sister to a clade containing *N. margaritacea* mtDNA as sister to all female-transmitted mt genomes of freshwater mussels. Minor differences with the nucleotide tree are as follows. In the female-transmitted mtDNAs clade: for Hyriidae, *W. carteri* F mtDNA is sister to all other hyriid female-transmitted mt genomes; for Unionidae, *Lanceolaria lanceolata* F mtDNA has a different position; in the Mulleriidae + Iridinidae clade, the positions of *C. rubens* and *M. dubia* are switched, with *M. dubia* here sister to all Mulleriidae (node posterior probability = 0.739). The M mt genomes clade differs from the nucleotide tree in the following instances: Margaritiferidae M mtDNAs have different relationships; in Unionidae, *Aculamprotula tortuosa* M mtDNA and *Schistodesmus* sp. [42] M mtDNA exchange positions, and *Sinanodonta woodiana* M mtDNA and *Anodonta anatina* M mtDNA become sister groups.

### Search and annotation of ORFan genes

**Fig. 3:**
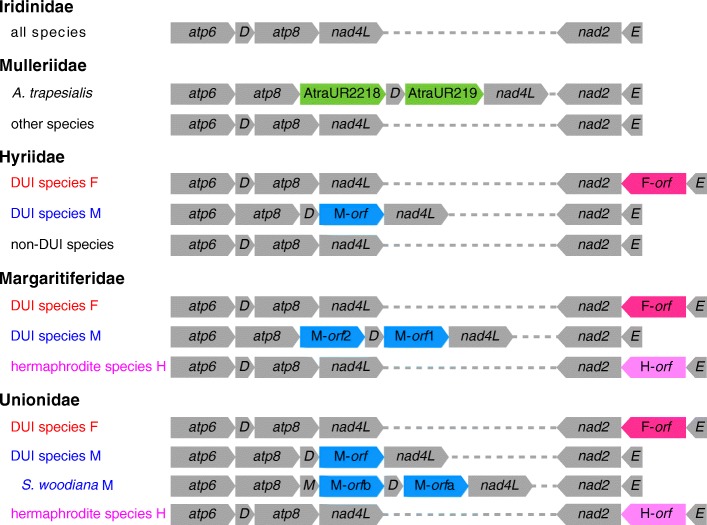
Schematic organization of the *atp6*-*nad4L* and *nad2*-*trnE* regions of the freshwater mussel (Unionida) based on mt genomes presented in this study and of already published ones. Sample size for each family: 2 Iridinidae (all non-DUI), 5 Mulleriidae (all non-DUI), 7 Hyriidae (2 F, 2 M, 3 non-DUI), 10 Margaritiferidae (6 F, 3 M, 1 H), 38 Unionidae (17 F, 17 M, 4 H). GenBank accession numbers of the mt genomes used are enlisted in Table 1 and Additional file 1: Table S1. Standard mitochondrial genes are in grey, while ORFan genes (see the main text for a complete description of these genes) are colored following this code: green, *Anodontites trapesialis* specific ORFans; blue, M-*orf*s; pink, F-*orf*s; light pink, H-*orf*s. Genes are pointed according to their relative direction on the mtDNAs. tRNA genes are indicated with the one-letter code of their respective amino acid. Dotted lines represent the segments between the two regions, which are not indicated for simplicity. *Sinanodonta woodiana* annotation is based on [12] and on the current study

To search for new ORFan genes, a set of 35,032 open reading frames (ORFs) (Table 4) was extracted from the new mt genomes in Table 1 and the additional 51 mtDNAs of freshwater mussels from GenBank (Additional file 1: Table S1), for a total of 62 mt genomes analyzed. Using as a criterion of choice the level of similarity between the known ORFan proteins and the translated proteins of the nucleotide sequences in the ORFs set, we found with the HMMER [43] suite of programs 25 F-*orf*s, 5 H-*orf*s, and 26 M-*orf*s, three of which are M-*orf*2 from Margaritiferidae species [5] and one appears to be a recent duplication specific to the unionid *S. woodiana* (we named the two copies M-*orf*a and M-*orf*b; see also [12]) (Additional file 1: Tables S2 and S3). The nucleotide sequences of these 56 ORFans are listed in Additional file 2. In summary, single ORFan protein sequences used as seeds mainly recognized homologous ORFan sequences, with few non-ORFan hits (as in the case for *E. menziesii* M-*ORF* which recognized some full, in-frame *nad4L* sequence) (Additional file 1: Table S2). The F-*ORF* Hidden Markov Model (HMM) profile we used recognized, in addition to all sequences from which it was assembled, also the H-*ORF*s (Additional file 1: Table S3). The M-*ORF* HMM profiles recognized all the M-*ORF*s forming them, plus, with lower scores and E-values, some protein translated from ORFs overlapping *trnD*, *atp8* (in two cases the hit comprised the whole in-frame sequence of its protein), *nad6*, and *nad4L* genes of many mt genomes (Additional file 1: Table S3). The putative proteins from the *AtraUR219* and *AtraUR2218* ORFans of *A. trapesialis*, located between *atp8* and *nad4L* of this species mt genome (Fig. 3) (which have been proposed to have originated from duplication and divergence of *atp8* and might be related to M-*orf*s of DUI species [5]), have no apparent homologs in other species. However, some ORFs from other species were retrieved that overlap the same region where the two *A. trapesialis* ORFans are located and from which are hypothesized to have originated: among these hits, for example, four include either a segment of the *atp8* gene or its full in-frame sequence (Additional file 1: Table S4). In short, as shown also in Fig. 3, we found that: (1) all DUI species of freshwater mussels analyzed carry a F-*orf* in their F mtDNA and at least one M-*orf* in their M; (2) secondarily hermaphrodite (i.e., that switched from gonochorism to hermaphroditism) species of Unionidae and Margaritiferidae that lost DUI always possess a H-*orf* [3]; (3) species that do not show evidence of DUI (i.e., no evidence of heteroplasmy) from families Iridinidae, Mulleriidae, and Hyriidae have none of these ORFans. The only mtDNA we retrieved from *P. auricularius* presents a standard F-*orf*, and given that this species is dioecious, it is plausible it will be revealed as a DUI species, and therefore we treated this genome as F mtDNA.

**Table 4:** ORFs dataset description

Family	Number of species	mtDNA type	Number of mtDNAs	Number of ORFs	F-*orf*	M-*orf*	H-*orf*
Iridinidae	2	non-DUI	2	1206	0	0	0
Mulleriidae	5	non-DUI	5	2820	0	0	0
Hyriidae	5	F	2	1063	2	0	0
		M	2	1099	0	2	0
		non-DUI	3	1668	0	0	0
		all	7	3830	2	2	0
Margaritiferidae	7	F	6	3273	6	0	0
		M	3	1657	0	6	0
		H	1	556	0	0	1
		all	10	5486	6	6	1
Unionidae	21	F	17	9698	17	0	0
		M	17	9826	0	18	0
		H	4	2166	0	0	4
		all	38	21,690	17	18	4
		all F	25	12,367	25	0	0
		all M	22	12,582	0	26	0
		all H	5	4389	0	0	5
		all non-DUI	10	5694	0	0	0
Total	40		62	35,032	25	26	5

For each taxonomic group of freshwater mussels, the number of species and mtDNAs considered in this study are given, as well as the number and kind of ORFans (F-, M-, or H-*orf*s) retrieved from them. mtDNA types: F and M, female- and male-transmitted mtDNAs of dioecious DUI species, respectively; H, mtDNA of stably hermaphroditic species that lost DUI sensu [3]; non-DUI, mtDNA of dioecious and hermaphroditic non-DUI species. mtDNAs of dioecious species carrying an F-*orf* but for which no evidence of DUI has been produced yet, or of dioecious DUI species that can show occasional hermaphroditism, were considered as F for simplicity

### Sequence-based analyses of ORFan protein products

**Fig. 4:**
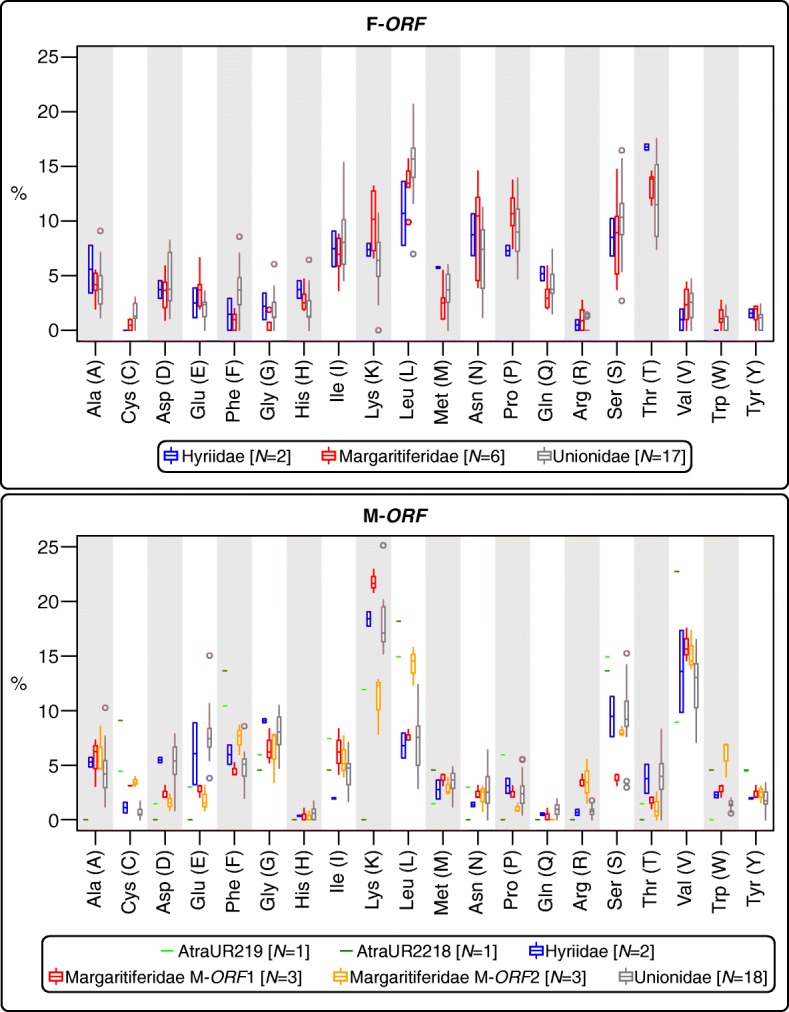
Percentage amino acid composition of the ORFan proteins considered in this study (their relative nucleotide sequences are enlisted in Additional file 2). The sample size for each boxplot is indicated inside the legends in square parentheses as ‘*N*’. Amino acid names are indicated with the IUPAC three-letter and one-letter codes

The amino acid composition of F-*ORF*s appears to be rather homogeneous among families, with no clear differences and, although more variable, a general trend is observable also in the M-*ORF*s (Fig. 4). For Margaritiferidae, in some cases, the distribution of some amino acid percentage of M-*ORF*2 proteins (which do not have a homolog in Hyriidae or Unionidae; Fig. 3), differ distinctly from the M-*ORF*1 and fall outside the range of other M-*ORF*s. The patterns for AtraUR219 is distinct from those of all M-*ORF*s but only in terms of sheer percentage of amino acid usage, as the peaks and declines of its pattern are located in the same position as the M-*ORF*s. On the contrary, AtraUR2218 profile is quite different and does not follow that of the other proteins; however, this may be an effect of its extremely short length (22 aa).

**Fig. 5:**
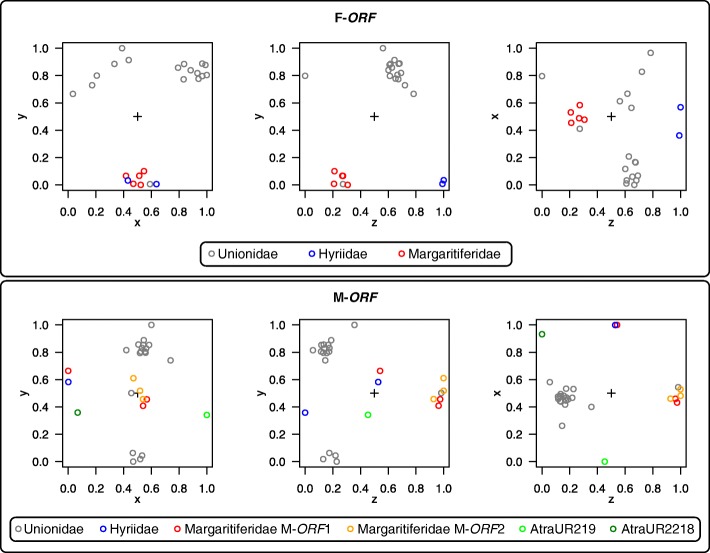
Summary of the CLANS analysis for F-*ORF*s and M-*ORF*s. Because the original CLANS output is a three-dimensional space, here are shown the three two-dimensional faces of the cube (one for each possible couple of axis: X vs Y, Z vs Y, Z vs X) obtainable by rotating the three-dimensional space of each analysis with 90° movements on one axis. The ‘+’ inside each panel represents the center of the cube. Each dot represents a single protein sequence (color code in the legends)

**Fig. 6:**
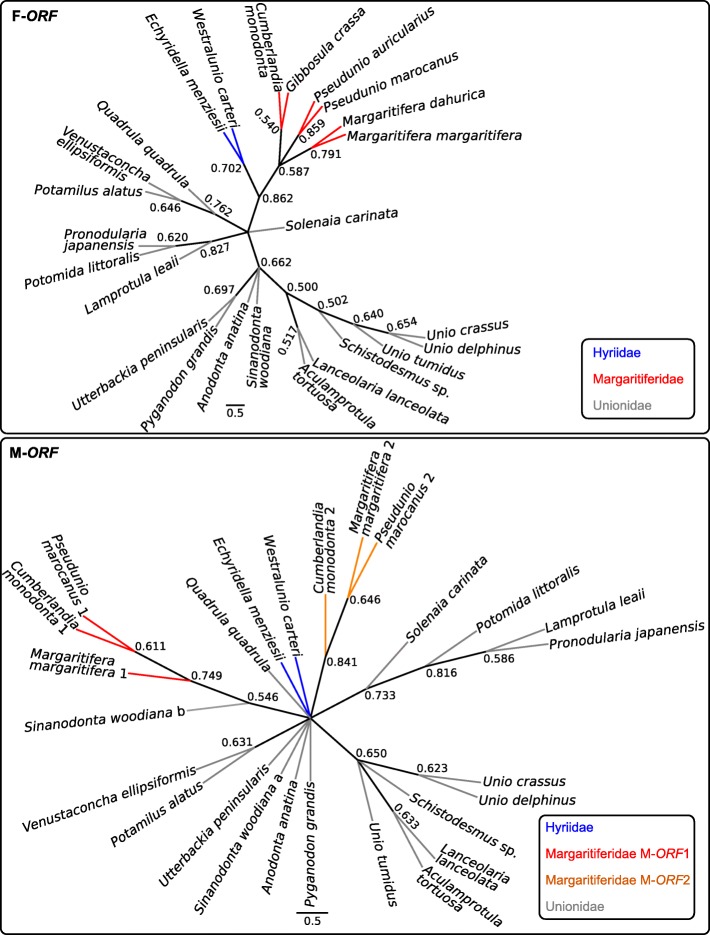
Unrooted maximum likelihood (ML) trees for F-*ORF* and M-*ORF* proteins of freshwater mussels. Color code for each family are indicated inside the panels. Bootstrap values are indicated at each node

Both the CLANS [44] analyses (Fig. 5) and the maximum likelihood (ML) trees (Fig. 6) tend to separate F-*ORF* and M-*ORF* protein sequences in family-specific clusters: notably, the relationships among single ORFans in the ML trees broadly resemble those among their respective mt genomes in our phylogenies based on 12 protein-coding genes (Figs. 1 and 2), especially in the case of F-*ORF*s. For Margaritiferidae M-*ORF*s, it is notable to see how M-*ORF*2 sequences cluster together with the M-*ORF*1 sequences in the CLANS analysis (Fig. 5), but form a separate branch in the ML tree (which has, however, low resolution) (Fig. 6). We also attempted to add AtraUR219 and AtraUR2218 sequences to the M-*ORF* alignment for the ML reconstruction, but this disrupted the clustering of M-*ORF*s, especially for the more numerous Unionidae (not shown).

### Tertiary structure prediction of ORFan proteins

**Fig. 7:**
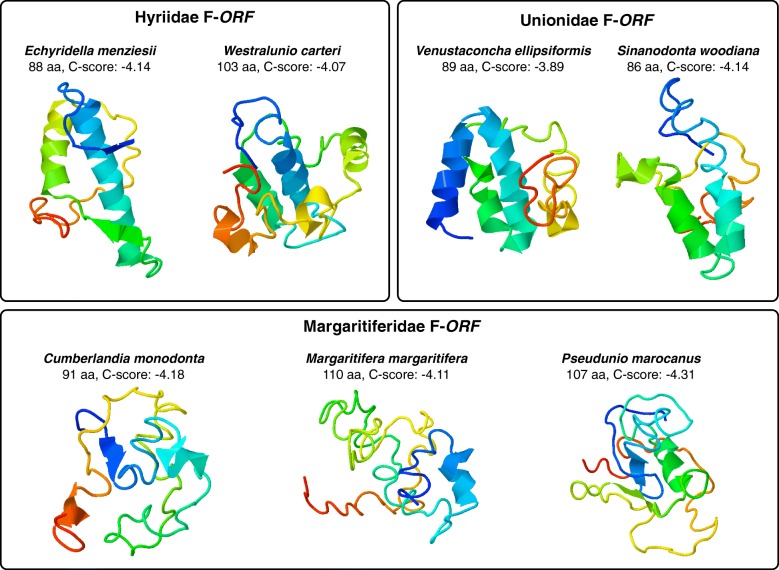
3D models of representative F-*ORF* proteins of DUI freshwater mussels. The models shown are the first of the top five predicted by I-TASSER for each sequence. Number of amino acids (aa) of each protein and C-score of the models are indicated under the relative species names. C-scores are usually comprised between − 5 and 2: higher values indicate higher confidence of the model. The color shading of each protein goes from the blue of the N-terminus to the red of the C-terminus

**Fig. 8:**
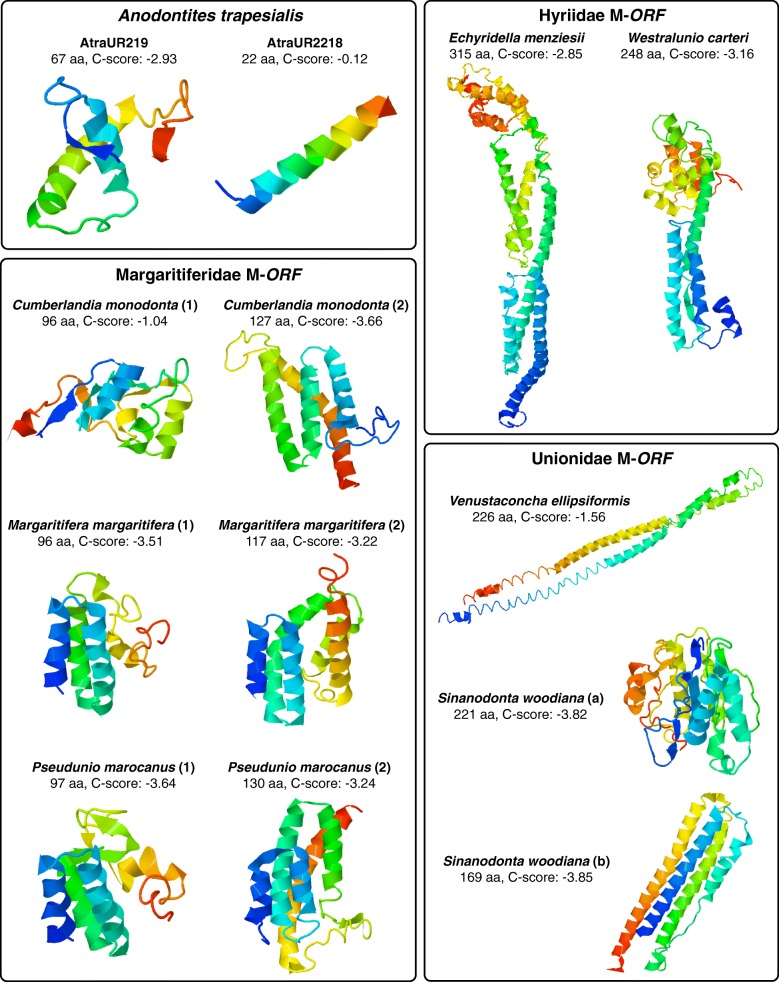
3D models of the proteins encoded by *Anodontites trapesialis* ORFans and of representative M-*ORF* proteins of DUI freshwater mussels. The models shown are the first of the top five predicted by I-TASSER for each sequence. Number of amino acids (aa) of each protein and C-score of the models are indicated under the relative species names. C-scores are usually comprised between − 5 and 2: higher values indicate higher confidence of the model. The color shading of each protein goes from the blue of the N-terminus to the red of the C-terminus

Currently, there are no data derived from crystallographic studies on the ORFan proteins, nor established structural similarities with known proteins in databases, that may guide bioinformatic analyses aimed at predicting the ORFan proteins folding. Therefore, for completeness, we decided to show the best results we obtained with I-Tasser [45] regardless of the C-scores assigned to the models: C-scores are usually comprised between − 5 and 2, therefore higher values indicate higher confidence of the model. The predicted tertiary structure of some selected ORFan proteins may appear to be highly different at first sight, but similarities among proteins of the same kind can be recognized (Figs. 7 and 8). A common feature between the F-*ORF*s of Hyriidae and Unionidae is the presence of two antiparallel helices separated by a loop. This conformation is not found in the F-*ORF* of Margaritiferidae, in which only one small helix is predicted (preceded by a small beta strand in *Cumberlandia monodonta* and *Pseudunio marocanus* but not in *Margaritifera margaritifera*). Hyriidae M-*ORF*, Margaritiferidae M-*ORF*1 and M-*ORF*2, and *S. woodiana* M-*ORF*a all share the presence of three antiparallel helices in their N-terminus. The three helices have the same relative orientation, but the third one is in front of the first two in Hyriidae and behind them in Margaritiferidae and *S. woodiana* (and it is also much smaller in this species). The portion beyond this third helix varies for each species, but, for example, Hyriidae M-*ORF*s are similar in this part of the protein and are clearly discernible from those of Margaritiferidae, and M-*ORF*1s and M-*ORF2*s of this family are again distinguishable between them. *Cumberlandia monodonta* M-*ORF*1 structure is less defined compared to the homologous proteins from *M. margaritifera* and *P. marocanus*, and in its M-*ORF*2, the third helix appears to be on the same plane as the other two. The three Unionidae M-*ORF*s examined have extremely divergent configurations, and no obvious similarities can be recognized among them. The two *S. woodiana* M-*ORF*s, most probably the product of a duplication event specific to this species [12], do not resemble one another. AtraUR219 protein is constituted by a short N-terminal beta strand, two helices crossing each other and connected by a simple loop, and a small C-terminal beta strand. AtraUR2218 protein is very short (22 aa) and it is predicted to be only a single helix.

### Three-dimensional alignments of ORFan proteins

Summarized statistics for pairwise three-dimensional (3D) alignments of the ORFan protein models are shown in Table 5. In the pairwise interspecies 3D alignment for each single freshwater mussel family, the distances (expressed as root mean square deviation of distances) between Cα and between Cß atoms (respectively the α- and ß-carbon atoms of an amino acid) tend to be lower in the M-*ORF* alignments (for Unionidae, only the *Venustaconcha ellipsiformis* M-*ORF* vs *S. woodiana* M-*ORF*a comparison) compared to the F-*ORF* ones, while percentages of aligned amino acids and secondary structures vary depending on the family and the ORFan proteins considered. Margaritiferidae M-*ORF*1 and − 2 pairwise alignments, both in between-species and in single-species comparisons, on average obtain relatively good (and in some cases better) values compared to the separate M-*ORF*1 and M-*ORF*2 alignments. The protein encoded by the recently duplicated M-*orf*b in *S. woodiana* appears to be rather distant in structure and sequence from the same species M-*ORF*a and *V. ellipsiformis* M-*ORF*. When aligning homologous ORFan proteins from different families, the best overall Cα and Cß atoms distance values and identity scores for the F-*ORF*s are those from the Unionidae versus Margaritiferidae comparisons. Hyriidae and Unionidae M-*ORF*s and Margaritiferidae M-*ORF*1 obtain similar, if not identical, Cα and Cß atoms distances results when compared among them in all combinations. These values are slightly higher when considering Margaritiferidae M-*ORF*2 sequences versus Hyriidae and Unionidae M-*ORF*s and, while the sequence identity of aligned amino acids tends to be higher for M-*ORF*1, the identity of aligned secondary structures is higher for M-*ORF*2 than that of M-*ORF*1 in the same comparisons.

**Table 5:** Summary statistics of the pairwise 3D alignments performed with MATRAS

Families	Proteins compared	N	Rdis (%)		RMS (Å)		DRMS (Å)		SqID (%)		Sec (%)	
			mean	SD	mean	SD	mean	SD	mean	SD	mean	SD
Hyriidae	F-*ORF*	1	4.50	n.a.	5.04	n.a.	3.38	n.a.	9.30	n.a.	46.50	n.a.
	M-*ORF*	1	1.00	n.a.	4.11	n.a.	2.07	n.a.	5.10	n.a.	100.00	n.a.
Margaritiferidae	F-*ORF*	3	7.50	4.16	8.98	4.13	5.11	0.68	13.20	7.28	92.43	7.92
	M-*ORF*1	3	10.90	17.33	3.58	1.92	2.58	1.21	20.87	23.86	63.97	13.95
	M-*ORF*2	3	9.67	7.11	5.35	3.84	3.53	2.29	14.23	5.66	83.37	5.70
	all M-*ORF*1 x M-*ORF*2	9	7.29	9.22	3.05	1.13	2.21	0.81	17.12	11.84	71.82	19.55
	single species M-*ORF*1 x M-*ORF*2	3	12.30	13.03	3.34	1.78	2.68	1.30	22.73	7.35	73.23	17.50
Unionidae	F-*ORF*	1	4.10	n.a.	4.18	n.a.	2.76	n.a.	5.40	n.a.	70.30	n.a.
	*V. ellipsiformis* M-*ORF* x *S. woodiana* M-*ORF*a	1	0.40	n.a.	2.36	n.a.	1.22	n.a.	0.00	n.a.	77.30	n.a.
	*V. ellipsiformis* M-*ORF* x *S. woodiana* M-*ORF*b	1	5.20	n.a.	35.75	n.a.	34.40	n.a.	9.20	n.a.	86.20	n.a.
	*S. woodiana* M-*ORF*a x M-*ORF*b	1	0.10	n.a.	3.75	n.a.	1.84	n.a.	0.00	n.a.	52.90	n.a.
Hyriidae x Margaritiferidae	F-*ORF*	6	0.08	0.21	2.79	1.50	2.33	0.92	3.97	6.26	66.83	43.03
	M-*ORF* x M-*ORF*1	6	0.45	0.28	3.50	3.40	1.85	1.62	8.77	9.03	76.22	16.18
	M-*ORF* x M-*ORF*2	6	2.10	1.47	4.74	2.24	2.62	0.60	8.17	4.72	84.40	11.79
Hyriidae x Unionidae	F-*ORF*	4	5.83	3.60	6.36	3.33	4.07	1.43	8.55	3.52	60.18	10.90
	M-*ORF*	6	1.30	1.54	3.50	0.90	1.92	0.57	7.65	3.57	70.77	7.06
Unionidae x Margaritiferidae	F-*ORF*	6	0.03	0.08	2.00	0.98	1.78	0.49	18.75	14.37	72.22	44.31
	M-*ORF* x M-*ORF*1	9	0.33	0.39	2.50	1.42	1.90	0.82	11.01	9.32	67.89	27.66
	M-*ORF* x M-*ORF*2	9	1.84	1.53	3.83	1.10	2.35	0.86	6.07	4.16	71.53	21.04

*N* Number of pairwise comparisons considered, *Rdis* Normalized Sdis score (%), *RMS* Root mean square deviation of Cα atoms location of aligned amino acids after optimal superimposition (Å), *DRMS* Root mean square deviation of distances between Cß atoms of aligned amino acids (Å), *SqID* % of identical amino acid pairs on the total number of aligned amino acids, *Sec* % of identical secondary structure residues on the total number of aligned amino acids, *SD* Standard deviation of the mean for a given value (n.a. when *N* = 1)

Multiple 3D alignments of ORFan proteins proved challenging for all groups we considered, i.e. all F-*ORF*s, all M-*ORF*s, and only Margaritiferidae M-*ORF*s (for the M-*ORF*s, we also tried to include the *A. trapesialis* proteins into the alignments). Only for a few combinations of sequences the alignment could successfully produce a tree but, because of the low number of sequences available, the trees did not show any informative clear-cut clustering patterns that could split the proteins into, for example, family-specific (e.g., Hyriidae vs Margaritiferidae vs Unionidae) or kind-specific (e.g., Margaritiferidae M-*ORF*1 vs M-*ORF*2) patterns as in the sequence-based analyses.

## Discussion

The deep relationships among freshwater mussel families have long been debated [46] but, although mt genomes from the sixth family Etheriidae are at present still not available, the phylogenies presented here support a sister group relationship between Hyriidae and Margaritiferidae + Unionidae, and an equally strict relationship between Mulleriidae and Iridinidae, although not well resolved: comparable family-level topologies were also found by other recent studies [5, 42, 46] using different analytical methods and/or taxa. Such topology supports an early classification by [47] that splits freshwater mussels in two superfamilies, Unionoidea (Unionidae + Margaritiferidae + Hyriidae) and Etherioidea (Mulleriidae + Iridinidae + Etheriidae). In the light of our and the other mentioned results [5, 42, 46], and as properly discussed by [46], the separation in these two major taxa better reflects the monophyly of shared characters among families than others that introduce a third superfamily, Hyrioidea, for Hyriidae only (as in [5]). It is worth noting how the female-transmitted mt genomes of Unionoidea, the superfamily where DUI is common, form a single clade sister to one containing mtDNAs of the non-DUI superfamily Etherioidea. This, however, does not imply that DUI was present only in the common ancestor of the Unionoidea. Indeed, the variable position of the M mtDNAs clade suggests the presence of DUI either in the last common ancestor of all freshwater mussels (Fig. 1) or even earlier, before the split between orders Unionida and Trigoniida, represented by the species *N. margaritacea* (Fig. 2). This is because when speciation occurs after DUI appears, F and M genomes evolve according to a “sex-associated” phylogenetic pattern [48] in two distinct clades and, inside these two clades, the relationships among mtDNAs of the various species are the same. Our phylogenies, therefore, suggest that (1) DUI was lost by Iridinidae and Mulleriidae, as well as by the South American lineage of Hyriidae (as discussed in detail below), and (2) that at least the last common ancestor of all Unionida had DUI. Comparable phylogenies were already retrieved with different methods and taxa [5, 42, 46] and, although the mt genomes sequenced in this study largely follow already described architectures [5, 29] (Fig. 3), we observed new interesting features that help us reconstruct the evolution of freshwater mussel mt genomes and their relationship with DUI.

Starting with family Mulleriidae, the four new mtDNAs have no trace of rearrangements that are reminiscent of the ORFans *AtraUR219* and *AtraUR2218* found in *A. trapesialis* (Table 2, Fig. 3), which were hypothesized to be early versions of M-*orf*s [5]. The structure of Mulleriidae mt genomes, therefore, makes them much more similar to Iridinidae mtDNAs, which also lack additional ORFans between *atp8* and *nad4L* (Table 2, Fig. 3). This can be interpreted as evidence for negative selection against the rise of novel coding sequences in both families Iridinidae and Mulleriidae. It is however notable how the two mentioned ORFans are present only in *A. trapesialis*, sister species to all other Mulleriidae in our phylogenies (Figs. 1, 2 and 3). Until further studies, this might indicate an independent and relatively recent genomic rearrangement in *A. trapesialis* giving rise to its two ORFans: therefore, future works investigating ORFans evolution should consider the possibility that both *AtraUR219* and *AtraUR2218* may be relicts of a species-specific duplication event, unrelated to M-*orf*s of other DUI families and possibly non-functional. Also, the selection against new sequences may not be correlated to the sexual system of these species, as both dioecious (*A. elongata*, *F. fossiculifera*, *C. rubens*) and hermaphroditic (*M. parchappi*) ones (Table 1) seem subject to it. For Iridinidae, *C. rubens* mtDNA also confirms that the non-coding region between *nad5* and *trnF* is unusually large compared to other freshwater mussel families [5]: whether it is a control region containing regulative motifs not found in other freshwater mussels will be a matter for future studies. Finally, the relationships among the examined Iridinidae and Mulleriidae species obtained from our phylogenies do not help in solving the history of their mt genome architectures. The two families, although strictly related, do not form separate monophyletic clades, and the two congeneric species *A. trapesialis* (hermaphrodite [39–41]) and *A. elongata* (dioecious [32]) are distantly related (Figs. 1 and 2): whether this situation calls for taxonomic revisions or not should be a matter for further ad hoc studies.

The new Margaritiferidae female-transmitted mt genome of *P. auricularius* has an F-*orf*, as previously described for this taxon [3, 5, 29] (Table 2, Fig. 3), and is strictly related to F mt genomes: further studies are surely needed to fully confirm the presence of DUI in this species, but the available evidence points to this direction. The re-analysis of published Unionidae mt genomes allowed us to confirm a recent species-specific duplication of the M-*orf* in *S. woodiana* M mtDNA, as noted by [12] (Fig. 3). We are unable to say what effects (if any) this mutation may have caused to *S. woodiana* DUI system, but our results suggest that the M-*orf*a (immediately upstream of *nad4L*) is the one more similar to those of other Unionidae, while M-*orf*b (immediately upstream of *trnD*) appears different, although still recognizable as an M-*orf*. This feature of *S. woodiana* and the previously described rearrangements in *A. trapesialis* support the idea that the *atp8*-*nad4L* region in the mtDNAs of freshwater mussels is a hotspot for significant rearrangements and gene duplications, therefore offering additional support to the hypothesis for which the unionid M-*orf* may have originated from a duplication of *atp8* [5, 28].

The mtDNAs of the only dioecious species of Hyriidae in this study showing DUI, *W. carteri*, are comparable in all aspects to those of the previously sequenced DUI species *E. menziesii* [5], a strictly related species with the same Australasian distribution. In particular, both the M-*orf* and the *cox2* in the M mtDNA are confirmed to be longer in this family compared to the others (Table 3). The other three Hyriidae species, *C. ambigua*, *D. suavidicus*, *P. obliquus* (all from South America and always forming a single monophyletic clade; Table 1, Figs. 1 and 2), did not show evidence of DUI and, contrary to DUI gonochoric and DUI-less hermaphroditic unionids and margaritiferids, their F-like mtDNAs do not possess any F-*orf* or H-*orf*, and we did not find evidence of their translocation in the unassigned regions of these mt genomes (Table 2, Fig. 3). Even if the sex determination system is known only for *C. ambigua*, a gonochoristic DUI-less species, we can propose a working hypothesis stating that, in Hyriidae, losing DUI (1) may always cause the complete disappearance of the F-*orf* in the former F mtDNA (as in *C. ambigua*, *D. suavidicus*, *P. obliquus*), and (2) may not affect the gonochoristic sexual system (as in *C. ambigua*). In this family, similarly to Mulleriidae and Iridinidae which are hypothesized to have lost DUI in the early stages of their radiation [5], it seems therefore that the relationship among DUI, presence of ORFans, and gonochorism may be somewhat different compared to Unionidae and Margaritiferidae, which retain a H-*orf* in their mt genomes after losing DUI [3]. The parallelism between Hyriidae and Mulleriidae + Iridinidae may, however, lead to another hypothesis: we can see that among all Hyriidae species studied until now, only the Australasian ones (*E. menziesii*, *W. carteri*) show DUI, while the Neotropical ones (*C. ambigua*, *D. suavidicus*, *P. obliquus*) do not (Table 1). This may hint that the last common ancestor for these two lineages had DUI, which was lost (together with the ORFan in the remaining F-like mtDNA) only in the South American lineage during its radiation. Considering the current information from all families of freshwater mussels, we can speculate that once DUI and the M mtDNA are lost by a species, the ORFan in the remaining F mtDNA (i.e. the F-*orf*) no longer plays a role in the DUI mechanism and gradually disappears. First, it may start to accumulate mutations and degenerate (like the H-*orf* in Unionidae and Margaritiferidae [3]) and then, given enough time, it completely disappears from the mtDNA without leaving recognizable traces (as in the South American Hyriidae, which no longer carry traces of the F-*orf*; Table 2, Fig. 3).

Despite very similar amino acid compositions (Fig. 4), sequence-based analyses of ORFan proteins managed in most cases to distinguish families (Figs. 5 and 6), with F-*ORF*s giving better resolution compared to M-*ORF*s (as in the ML analysis, for example). Following this lead, we explored for the first time the total putative 3D folding of freshwater mussels ORFan proteins (as secondary structures and other features have been already thoroughly characterized [27, 28, 49]), to search for patterns that could help unravel their evolutionary history. Indeed, even if we sampled only a few representative species, the predicted 3D foldings demonstrate how in each family the F- and M-*ORF*s have their own peculiar shape (Figs. 7 and 8). The tertiary structure of a protein is influenced by its amino acid sequence, and as a consequence, the proteins of closely related species and/or with a common origin exhibit comparable shapes. In Margaritiferidae, for example, all M-*ORF*1 and M-*ORF*2 proteins fold in a similar way, and this finding could also hint at a full functionality of the M-*ORF*2 protein in DUI margaritiferids, possibly with a physiological role comparable to that of M-*ORF*1 and other M-*ORF*s. In contrast, in the case of Unionidae M-*ORF*s (Fig. 8), the proteins exhibit a range of very divergent foldings; this could be due to the method used, the phylogenetic distance among species (see their positions in the trees in Figs. 1 and 2), or the exclusive evolutionary history of each protein (as we discussed above for *S. woodiana* M-*ORF*s). A broader sampling from each family and a comparison among different methods of tertiary structure prediction in the future could help to better define these ambiguous foldings, as well as to improve the distance analyses that lacked resolution in the current study.

Nonetheless, despite the technical difficulties, our explorative study presents evidence of possibly conserved features among ORFan proteins of the same kind, such as the relative arrangement of certain helices in F-*ORF*s and M-*ORF*s. These structural features, together with properties already characterized (e.g., [5, 27, 28, 49]) and others yet to be discovered, will lead us to give a precise physiological role to the ORFan proteins and their respective genes (like those already hypothesized before, for example [28]). With further study, we might also be able to answer the long-standing questions about the relationships among the ORFan genes, the sex determination system, and the peculiar mitochondrial inheritance mode of freshwater mussels and of all other bivalves showing DUI [3]. However, given the rather different length and shape of Hyriidae M-*ORF*s compared to those of Unionidae and Margaritiferidae, and the fact that Hyriidae DUI-less species are not always hermaphroditic (as *C. ambigua*) and their mtDNA does not possess any F- or H-*orf* (*C. ambigua*, *D. suavidicus*, *P. obliquus*), as opposed to what occurs in Margaritiferidae and Unionidae [3], we suspect that the nature of the link among ORFans and sexual system may not be exactly the same in all DUI freshwater mussels. On the other hand, it is also possible that long evolutionary times in the absence of DUI, as well as various ecological pressures [50], may shape freshwater mussel mt genomes and/or sexual systems, leading to the situations described for the first time in this study (i.e., no ORFan genes in both hermaphroditic and gonochoric species). To answer these questions, a broader sampling and in vivo studies on the ORFan proteins will be needed.

## Conclusion

In this study we produced ten entire, plus one almost complete, new mtDNA sequences of freshwater mussel species with or without DUI from still poorly sampled families (Iridinidae, Mulleriidae, Hyriidae, and Margaritiferidae). Besides being a useful basis for future sequencing efforts, much needed for this group of endangered animals [51], we provided new data on the mitochondrial ORFan genes of freshwater mussels, whose origin is still unknown and whose function and conservation are likely related to their sex determination system [3]. We observed that the rearrangements occurring in mitochondrial genomes of species and lineages that secondarily lost DUI (i.e., that lost their ancestral M mtDNA and retain only a mutated F) are not always the same, and that losing DUI is not always linked to a switch to hermaphroditism. By analyzing the 3D structures of their translated proteins, we also evidenced common characteristics and similarities among them, hinting at conserved physiological roles of F-*orf* and M-*orf* genes in all DUI lineages of freshwater mussels, as well as family-specific ones. We therefore questioned if the family-specific structures of the ORFan proteins can influence some detail of the DUI system in different manners, so that the downstream effect of losing DUI on the sexual system of a species may vary. An alternative, but not necessarily mutually exclusive, hypothesis we propose to explain the observed differences among non-DUI lineages is that time and other factors may play an important role in reshaping both the mitochondrial genome and sexual system of a species after it loses DUI.

## Methods

### Sequencing of new mt genomes

Freshwater mussel species were selected across the main families within the order Unionida and to cover distinct sexual strategies, i.e. gonochorism and hermaphroditism (Table 1). *W. carteri* specimens were taken from the wild with permits for field and laboratory studies obtained from the Western Australian Department of Environment and Conservation under Regulation 17 of the Wildlife Conservation Act 1950 (SF007049) and Department of Fisheries under Exemption from the Fisheries Resources Management Act 1994 (1724–2010-06). Sex of the specimens was determined by observing gonad tissue smears for sexual cells and/or the demibranchs for the presence of marsupia, using a dissecting microscope. Tissue samples were excised from one specimen per species, and placed in 100% ethanol for DNA extraction: for all species, a foot clip was available for DNA extraction, while for *W. carteri* an additional male gonad sample from the same specimen was also used. DNA extraction followed [52]. DUI presence or absence for every species was assumed from previous studies [30–37]. The complete mitogenomes sequencing and assemblage was accomplished using the pipeline proposed by [53]. Annotations were performed using MITOS [54] with the final tRNA genes limits being rechecked with ARWEN [55]. Finally, personal scripts were developed and applied to adjust the mtDNA protein-coding limits since MITOS seems to underestimate gene length (for details, go to https://figshare.com/s/a756ef19cec8f65d506a).

### Phylogenetic analyses of freshwater mussel mt genomes

The set of eleven new mt genomes (Table 1) was expanded by adding other 51 freshwater mussel mt genomes from GenBank (see Additional file 1: Table S1 for the complete list and details), for a total of 62 mtDNAs for 40 species. The mt genomes added encompass families Iridinidae, Mulleriidae, Hyriidae, Margaritiferidae, and Unionidae. For Hyriidae and Unionidae, we took the mt genomes from DUI species for which both the F and M mtDNAs were available (resulting in two mtDNAs per species), for Margaritiferidae all F and M mt genomes available, and for Margaritiferidae and Unionidae only also those from secondarily hermaphrodite species (i.e., the H mtDNAs). The final set was thus composed of 22 M, 25 F, and 15 mt genomes from non-DUI species, either secondarily hermaphrodite that lost DUI (sensu [3]) or gonochoric ones. For the purpose of the phylogenetic analyses, three outgroup species mt genomes were also used: the bivalves *Neotrigonia margaritacea* (Trigoniida) and *Solemya velum* (Solemyida), plus *Chaetoderma nitidulum* (Caudofoveata, Chaetodermatida) as outgroup to all bivalves (respective GenBank accession numbers: KU873118, JQ728447, EF211990). A total of 65 mt genomes was therefore considered for the phylogenies.

We extracted all protein coding gene sequences, except *atp8* because of its short length and high variability, from the 65 mtDNAs and translated them with the invertebrate mitochondrial genetic code to obtain the relative protein sequences. The 12 protein sets were then aligned with M-Coffee (http://tcoffee.crg.cat/apps/tcoffee/do:mcoffee) [56, 57] using all multiple methods available, then from these protein alignments we retro-aligned the codons of the respective genes using the TranslatorX server (http://translatorx.co.uk) [58]. Both protein and codon alignments were trimmed on the Gblocks server version 0.91b (http://molevol.cmima.csic.es/castresana/Gblocks_server.html) [59, 60] using the option for a more stringent selection. jModelTest2 [61, 62] was used to calculate, under the Bayesian Inference Criterion (BIC), the best-fit models of nucleotide substitution for the trimmed codon alignments. Finally, the two sets of trimmed alignments were concatenated respectively into a codon alignment and an amino acid alignment, with a respective length of 7914 and 2363 gapless positions. MrBayes 3.2.3 [38] was used to infer amino acid- and nucleotide-base phylogenies of the mt genomes. The analysis for both concatenated alignments consisted of two separate runs of four chains, 5,000,000 generations, sampling every 100 trees with a burn-in of 0.1%. In the nucleotide analysis, the models retrieved with jModelTest2 were specified for each gene partition, and a ‘4by4’ nucleotide substitution model was adopted for the whole alignment. In the amino acid analysis, a ‘mixed’ rate matrix was specified. Completed runs were accepted for further examination after checking that their standard deviation of split frequencies stabilized at values < 0.01 over the generations (as in [63]). jModelTest2 and MrBayes 3.2.3 were ran on the CIPRES Science Gateway (http://www.phylo.org) [64]. Trees were graphically edited with FigTree v.1.4.3 [65].

### Annotation of F- and M-*orf* genes

To locate the F- and M-*orf* genes in the DUI genomes in which they were not annotated, and at the same time validate previous annotations, we first used the EMBOSS tool getorf [66] to extract all possible ORFs ≥30 nucleotides long (i.e., coding at least 10 codons) under the invertebrate mitochondrial genetic code from the 62 freshwater mussel mtDNAs dataset described above, and then translated them into the corresponding proteins. This set of protein sequences was first searched with the HMMER tool jackhmmer [43] (10 iterations) using as seeds the F-*ORF* of *V. ellipsiformis* and the M-*ORF*s of *E. menziesii*, *C. monodonta* (M-*ORF*1) and *V. ellipsiformis*, in separate runs. The proteins retrieved from each run were then aligned with PSI-Coffee [56, 67] (http://tcoffee.crg.cat/apps/tcoffee/do:psicoffee; all pairwise methods selected), and the alignments used to build HMM profiles with hmmbuild [43] (options: -fast -symfrac 0 -fragthresh 0 -wnone -enone; see also [28]). These profiles were used to search again the whole set of proteins with hmmsearch [43] (−-max option active to allow maximum sensitivity) to confirm the presence of F-*orf*s and M-*orf*s previously found with jackhmmer, retrieve the known ones not recognized by jackhmmer, and search for new homologs of these genes. Finally, phmmer [43] (−-max option active) was used to search the protein set for homologs of the two proteins putatively encoded by the two ORFans *AtraUR219* and *AtraUR2218* in *A. trapesialis* (Mulleriidae) mtDNA, hypothesized to be related to M-*orf* genes [5].

### Sequence-based analyses of proteins

MEGA7 [68] was used to calculate the amino acid composition of F-*ORFs*, M-*ORFs*, and AtraUR219 and AtraUR2218 of *A. trapesialis*. To visualize the relationships among the already annotated and newly discovered ORFan proteins based on pairwise similarity, we ran CLANS [44]. Specifically, the CLANS program was conducted online on the MPI Bioinformatic Toolkit website [69] (https://toolkit.tuebingen.mpg.de/#/tools/clans) using the BLOSUM45 scoring matrix and BLAST HSP’s E-values up to 1E-4. The output was then run locally on the CLANS application for ≥10,000,000 rounds to obtain reliable 3D clustering data. The alignments of the F-*ORF*s and M-*ORF*s used to build their HMM models, plus an alignment of the two *A. trapesialis* ORFan proteins and the M-*ORF*s (constructed with PSI-Coffee as mentioned above), were used to build ML trees in MEGA7 [68], using 1000 bootstraps, the ‘mtREV’ model of substitution, uniform rates among sites, and a gap partial deletion of 95%. The trees were built unrooted because given the uncertain origin of the ORFan genes it is not possible to choose a reliable outgroup sequence.

### 3D structure-based analyses of proteins

We used I-TASSER [45] online (https://zhanglab.ccmb.med.umich.edu/I-TASSER/) to obtain the 3D models of the F- and M-*ORF* of some representative DUI species (Hyriidae: *E. menziesii*, *W. carteri*; Margaritiferidae: *C. monodonta*, *M. margaritifera*, *P. marocanus*; Unionidae: *V. ellipsiformis*, *S. woodiana*) plus AtraUR219 and AtraUR2218 of *A. trapesialis* (Mulleriidae). The most supported models (i.e., the ones with the best C-score) were then used as input for MATRAS [70] (http://strcomp.protein.osaka-u.ac.jp/matras/) to perform pairwise and multiple 3D alignments of the proteins. The multiple alignments aimed at obtaining trees based on DRMS (root mean square deviation of Cα atoms, measured in Å) distances among them, using as a minimal set the ORFan proteins from *E. menziesii*, *C. monodonta*, and *V. ellipsiformis* (which have been thoroughly characterized in past studies [3, 5, 27, 28]) and adding as much proteins as MATRAS would allow from the other species. When an I-TASSER model made MATRAS fail in producing a tree, we refined it with ModRefiner [71] (https://zhanglab.ccmb.med.umich.edu/ModRefiner/) and repeated the 3D alignment. If the refining did not succeed in improving the results, the protein was removed from the analysis.

## Supplementary information

**Additional file 1: Table S1.** Summary of the additional 51 freshwater mussel mt genomes used in this study that were retrieved from GenBank. **Table S2.** Summary of the jackhmmer analyses (F-*ORF* and M-*ORF* sequences input vs protein set). **Table S3.** Summary of the hmmsearch analyses (F-*ORF* and M-*ORF* HMM profiles input vs protein set). **Table S4.** Summary of the phmmer analyses (*A. trapesialis* ORFan proteins input vs protein set).

**Additional file 2.** List of mitochondrial ORFan gene sequences of freshwater mussels whose translated protein products were used in this study.

## Data Availability

The mtDNA sequences obtained in this study have been submitted to GenBank under the accession numbers MK761136–46.

## Abbreviations

3D: Three-dimensional
aa: Amino acid
*atp8*: ATP synthase F0 subunit 8 gene
*AtraUR219*, *AtraUR2218*: ORFan genes found in *Anodontites trapesialis* (Mulleriidae) mtDNA hypothesized to be related to the M-*orf* in the M mtDNA of DUI freshwater mussels
AtraUR219, AtraUR2218: Protein products of *AtraUR219* and *AtraUR2218*, respectively
BIC: Bayesian Information Criterion
*cox2*: Cytochrome c oxidase subunit 2 gene
Cß: ß-carbon atom of an amino acid, located right before the α-carbon
C-terminus: End of a protein chain
Cα: α-carbon atom of an amino acid, located right before the carbonyl carbon
DUI: Doubly uniparental inheritance (of mitochondria)
F-*orf*: ORFan gene typically found in the F mtDNA of freshwater mussels showing DUI
F-*ORF*: Protein product of a F-*orf* gene
HMM: Hidden Markov Model
H-*orf*: ORFan gene typically found in the mtDNA of strictly hermaphroditic Unionidae and Margaritiferidae species that secondarily lost DUI, it is a mutated version of a F-*orf*
H-*ORF*: Protein product of a H-*orf* gene
ML: Maximum likelihood
M-*orf*: ORFan gene typically found in the M mtDNA of freshwater mussels showing DUI, usually found in single-copy but present in two copies in Margaritiferidae (named 1 and 2) and in *Sinanodonta woodiana* (Unionidae) (named a and b)
M-*ORF*: Protein product of a M-*orf* gene
mt: Mitochondrial
mtDNA: Mitochondrial DNA
*nad2*, *− 4 L*, − *5*, *6*: NADH dehydrogenase subunits 2, 4 L, 5, 6 genes, respectively
N-terminus: Start of a protein chain
ORF: Open reading frame
ORFan: ORF with no recognizable homology or similarity to known genes
tRNA: Transfer RNA
*trnD*, *−E*, −*F*, −*H*, −*V*: Genes encoding tRNAs respectively for aspartic acid, glutamic acid, phenylalanine, histidine, valine
