# Supplementary material for: Variability of mitochondrial ORFans hints at possible differences in the system of doubly uniparental inheritance of mitochondria among families of freshwater mussels (Bivalvia: Unionida)
Source: BMC Evol Biol. 2019 Dec 19;19:229. doi: 10.1186/s12862-019-1554-5 (PMC6923999; doi:10.1186/s12862-019-1554-5)
Supplement: Supplementary file 1 — Additional file 1: Table S1. Summary of the additional 51 freshwater mussel mt genomes used in this study that were retrieved from GenBank. Table S2. Summary of the jackhmmer analyses (F-ORF and M-ORF sequences input vs protein set). Table S3. Summary of the hmmsearch analyses (F-ORF and M-ORF HMM profiles input vs protein set). Table S4. Summary of the phmmer analyses (A. trapesialis ORFan proteins input vs protein set). [file 12862_2019_1554_MOESM1_ESM.pdf]

ADDITIONAL FILE 1

Table S1

Details for the additional mt genomes and of their respective freshwater mussel species used in this study. The corresponding working acronym used throughout the study for each species is indicated. For each mtDNA, its GenBank accession number and type are specified: non-DUI, mtDNA of species without evidence of DUI presence or secondary loss; F and M, female- and male-transmitted mtDNAs of dioecious DUI species, respectively; H, mtDNA of a secondarily hermaphroditic species (derived from an ancestral F).

The species acronyms MarCra, MarMrc, LamTor, ArcLan, UniJap, and LamGot appearing in this table stand for the working names of the species used during elaboration of the data, i.e. *Margaritifera crassa*, *Margaritifera marocana*, *Lamprotula tortuosa*, *Arconaia lanceolata*, *Unio japanensis*, and *Lamprotula gottschei*, which were respectively updated during the writing of this article to the more correct *Gibbosula crassa*, *Pseudunio marocanus*, *Aculamprotula tortuosa*, *Lanceolaria lanceolata*, *Pronodularia japanensis*, and *Schistodesmus* sp.. These six mentioned acronyms appear also in the other tables of this file.

			**GenBank accession numbers**			
**Family**	**Species**	**Acronym**	**non-DUI**	**F**	**M**	**H**
Iridinidae	*Mutela dubia*	MutDub	KU873120			
Mulleriidae	*Anodontites trapesialis*	AnoTra	KU873119			
Hyriidae	*Echyridella menziesii*	EchMen		KU873121	KU873122	
Margaritiferidae	*Cumberlandia monodonta*	CumMon		KU873123	KU873124	
	Gibbosula crassa	MarCra		MH319826		
	Margaritifera dahurica	MarDah		KF514426		
	Margaritifera falcata	MarFal				HM856634
	Margaritifera margaritifera	MarMar		MK421956	MK421959	
	Pseudunio marocanus	MarMrc		KY131953	KY131954	
Unionidae	*Aculamprotula tortuosa*	LamTor		KC109779	KC441487	
	Anodonta anatina	AnoAna		KF030964	KF030962	
	Lamprotula leaii	LamLea		JQ691662	KC847114	
	Lanceolaria lanceolata	ArcLan		KJ144818	KJ775864	
	Lasmigona compressa	LasCom				HM856638
	Lasmigona subviridis	LasSub				HM856640
	Potamilus alatus	PotAla		KU559011	KU559010	
	Potomida littoralis	PotLit		KT247374	KT247375	
	Pronodularia japanensis	UniJap		AB055625	AB055624	
	Pyganodon grandis	PygGra		FJ809754	FJ809755	
	Quadrula quadrula	QuaQua		FJ809750	FJ809751	
	Schistodesmus sp.	LamGot		KJ018924	KJ627225	
	Sinanodonta woodiana	SinWoo		KM272949	KM434235	
	Solenaia carinata	SolCar		KC848654	KC848655	
	Toxolasma parvum	ToxPar				HM856639
	Unio crassus	UniCra		KY290446	KY290450	
	Unio delphinus	UniDel		KT326917	KT326918	
	Unio tumidus	UniTum		KY021077	KY021073	
	Utterbackia imbecillis	UttImb				HM856637
	Utterbackia peninsularis	UttPen		HM856636	HM856635	
	Venustaconcha ellipsiformis	VenEll		FJ809753	FJ809752	

Table S2

Summary of the jackhmmer analyses. The input sequences (‘Query Name’) of the single runs were the protein sequences translated from the F-*orf* of *Venustaconcha ellipsiformis* and the M-*orf*s of *Echyridella menziesii*, *Cumberlandia monodonta* (M-*orf*1) and *V. ellipsiformis*, which are enlisted in Additional file 2. For each sequence identified (‘Target Name’) in the set of 35,032 ORFs extracted from all considered mtDNAs with getorf, are shown the full sequence statistics (‘E-value’, ‘Score’, ‘Bias’) taken from the ‘per target’ output produced by jackhmmer with a short description of the sequence. Apart from some low-quality hits, no previously unrecognized F-*orf* and M-*orf* sequences were identified at this stage.

Species acronyms for the new mt genomes sequenced in this study appearing in the column ‘Target Name’: ChaRub for *Chambardia rubens*, AnoElo for *Anodontites elongata*, FosFos for *Fossula fossiculifera*, LamEns for *Lamproscapha ensiformis*, MonPar for *Monocondylaea parchappii*, CasAmb for *Castalia ambigua*, DipSua for *Diplodon suavidicus*, PriObl for *Prisodon obliquus*, WesCar for *Westralunio carteri*, MarAur for *Pseudunio auricularius* (formerly *Margaritifera auricularia*, used during data elaboration). Other acronyms and their respective species names are enlisted in Table S1.

Query Name	Target Name	E-value	Score	Bias	Description
u_VenEllF_FORF	u_LasSubH_257	1.3e-53	180.5	19.6	corresponds to H-*orf*, overlaps with upstream *trnE*
	u_LasComH_332	2.3e-36	125.2	24.4	corresponds to H-*orf*
	u_VenEllF_322	1e-29	103.9	11.5	corresponds to F-*orf*, overlaps with upstream *trnE* and *trnS*
	u_PotAlaF_317	6e-27	95.0	11.0	corresponds to F-*orf*, overlaps with upstream unassigned region
	m_MarDahF_328	1.4e-23	84.2	11.0	corresponds to F-*orf*, overlaps with upstream unassigned region
	u_QuaQuaF_339	8.6e-23	81.7	7.5	corresponds to F-*orf*
	m_MarFalH_322	1.2e-20	74.8	21.2	corresponds to H-*orf*
	m_MarAurF_317	1.2e-20	74.7	9.1	corresponds to F-*orf*
	m_CumMonF_319	2.6e-19	70.5	13.0	corresponds to F-*orf*, overlaps with upstream *trnE* and its 5’ unassigned region
	u_LamGotF_340	6e-19	69.3	1.4	corresponds to F-*orf*
	m_MarMrcF_320	9.2e-19	68.8	9.1	corresponds to F-*orf*
	u_LamLeaF_338	1.1e-18	68.5	9.2	corresponds to F-*orf*, overlaps with upstream *trnE*
	u_UniCraF_334	2.1e-18	67.6	5.8	corresponds to F-*orf*
	m_MarMarF_298	1.3e-17	65.1	9.5	corresponds to F-*orf*, overlaps with upstream unassigned region
	u_UniTumF_325	2.4e-17	64.2	10.3	corresponds to F-*orf*, overlaps with upstream *trnE*
	u_UniDelF_344	5.5e-17	63.1	7.3	corresponds to F-*orf*
	u_PotLitF_316	2e-16	61.3	3.5	corresponds to F-*orf*, overlaps with upstream *trnE*
	u_AnoAnaF_353	2.2e-16	61.2	7.0	corresponds to F-*orf*
	u_PygGraF_310	1.6e-15	58.3	2.3	corresponds to F-*orf*
	u_UttPenF_329	8.3e-15	56.1	0.7	corresponds to F-*orf*
	u_LamTorF_317	1.4e-14	55.4	5.3	corresponds to F-*orf*
	u_SinWooF_343	1.4e-14	55.4	10.0	corresponds to F-*orf*
	u_UniJapF_340	2.3e-14	54.7	2.3	corresponds to F-*orf*, overlaps with upstream *trnE*
	u_ArcLanF_342	3.8e-14	54.0	9.1	corresponds to F-*orf*, overlaps with upstream *trnE*
	h_EchMenF_311	3.8e-14	53.9	14.6	corresponds to F-*orf*, overlaps with upstream *trnE*
	u_SolCarF_363	1.3e-13	52.3	5.4	corresponds to F-*orf*, overlaps with downstream *nad2* and upstream *trnE* and *trnS*
	m_MarCraF_334	1.3e-12	49.1	11.0	corresponds to F-*orf*
	u_ToxParH_333	3.6e-06	28.4	3.4	corresponds to H-*orf*, overlaps with downstream *nad2* and upstream *trnE* and *trnS*
	h_WesCarF_330	0.0012	20.3	17.5	corresponds to F-*orf*, overlaps with upstream *trnE*
	u_UttImbH_346	0.05	15.1	0.1	corresponds to H-*orf*, overlaps with upstream *trnE* and its 5’ unassigned region
	h_WesCarF_518	0.32	12.5	4.2	inside *cox3* (opposite coding direction)
h_EchMenM_MORF	h_EchMenM_55	3.1e-193	642.1	31.0	corresponds to M-*orf*, overlaps with upstream *trnD*
	h_WesCarM_56	5.7e-101	339.0	30.9	corresponds to M-*orf*, overlaps with upstream *trnD*
	u_UniJapM_65	0.15	12.4	7.2	corresponds to *nad4L*
	u_LamLeaM_555	0.33	11.2	4.0	inside *cox1* (opposite coding direction)
	h_EchMenF_57	0.98	9.7	2.9	corresponds to *nad4L*
	i_ChaRub_57	5.8	7.1	5.7	corresponds to *nad4L*
	y_MonParF_62	6.1	7.0	4.2	corresponds to *nad4L*
	y_FosFosF_66	7.1	6.8	4.4	corresponds to *nad4L*
	m_MarMrcM_141	7.3	6.8	4.8	inside *nad5* (same coding direction, different frame)
m_CumMonM_MORF1	m_CumMonM_64	8.70e-49	164.8	14.8	corresponds to M-*orf*1
	m_MarMrcM_71	6.50e-46	155.6	15.3	corresponds to M-*orf*1, overlaps upstream *trnD* and its 5’ unassigned region
	m_MarMarM_78	4.20e-44	149.8	14.3	corresponds to M-*orf*1
	m_MarMarM_70	3.50e-29	102.0	11.3	corresponds to M-*orf*2
	m_MarMrcM_62	4.70e-24	85.6	14.4	corresponds to M-*orf*2
	m_CumMonM_56	9.00e-23	81.5	5.9	corresponds to M-*orf*2
	u_PygGraF_248	0.29	12.5	2.6	overlaps F-*orf* and *trnE* (opposite coding directions)
	u_SolCarM_58	2	9.9	8.0	corresponds to M-*orf*, overlaps upstream unassigned region
u_VenEllM_MORF	u_VenEllM_59	1.60e-118	395.3	36.2	corresponds to M-*orf*
	u_PygGraM_64	2e-79	267.5	18.1	corresponds to M-*orf*

Query Name	Target Name	E-value	Score	Bias	Description
	u_PotAlaM_63	4.2e-38	132.3	13.3	corresponds to M-*orf*
	u_LamTorM_61	2.4e-27	97.1	34.7	corresponds to M-*orf*
	u_LamGotM_63	9.5e-27	95.1	30.7	corresponds to M-*orf*
	u_AnoAnaM_62	5.2e-26	92.7	25.4	corresponds to M-*orf*
u_VenEllM_MORF	u_ArcLanM_55	9.4e-26	91.9	28.7	corresponds to M-*orf*
	u_UniCraM_62	6e-25	89.2	32.0	corresponds to M-*orf*, overlaps upstream unassigned region
	u_UniTumM_61	1e-24	88.5	22.5	corresponds to M-*orf*
	u_UttPenM_56	5.9e-23	82.7	24.4	corresponds to M-*orf*, overlaps upstream unassigned region
	u_UniDelM_58	6.8e-22	79.3	3.7	corresponds to M-*orf*, overlaps upstream *trnD*
	u_QuaQuaM_67	3e-20	73.9	9.3	corresponds to M-*orf*, overlaps upstream *trnD* and downstream *nad4L*
	u_SinWooM_77	1.8e-16	61.5	21.5	corresponds to M-*orf*a

Table S3

Summary of the hmmsearch analyses. The HMM profiles used were composed of all considered protein sequences of F-*ORF*s (hmm_FORF, 25 sequences), M-*ORF*s and M-*ORF*1s (hmm_MORF01, 23 sequences), M-*ORF*2s (hmm_MORF02, 3 sequences), and all kind of M-*ORF*s together (hmm_MORF12, 26 sequences). Some known M-*ORF* sequences not previously found with jackhmmer were still included in the respective profiles. For each sequence identified ('Sequence') in the set of 35,032 ORFs extracted from all considered mtDNAs with getorf, are shown the full sequence statistics ('E-value', 'Score', 'Bias') taken from the output produced by hmmsearch. Hits below the inclusion threshold of hmmsearch are separated by a dotted line in the table. In the 'Description' column, a * indicates a protein sequence included in the HMM profile used, and a short description of the sequence is given only for hits not already found with jackhmmer (i.e., hits with no description have been already described in Table S2).
Species acronyms for the new mt genomes sequenced in this study appearing in the column 'Target Name': ChaRub for *Chambardia rubens*, AnoElo for *Anodontites elongata*, FosFos for *Fossula fossiculifera*, LamEns for *Lamproscapha ensiformis*, MonPar for *Monocondylaea parchappii*, CasAmb for *Castalia ambigua*, DipSua for *Diplodon suavidicus*, PriObl for *Prisodon obliquus*, WesCar for *Westralunio carteri*, MarCra for *Pseudunio auricularius* (formerly *Margaritifera auricularia*, used during data elaboration). Other acronyms and their respective species names are enlisted in Table S1.

HMM Profile	Sequence	E-value	Score	Bias	Description
hmm_FORF	m_MarAurF_317	1.90e-65	223.0	24.1	*
	u_LasSubH_257	3.10e-47	162.9	23.4	
	m_MarMarF_298	1.80e-44	153.9	18.6	*
	m_MarMrcF_320	3.10e-42	146.5	21.3	*
	m_MarDahF_328	7.50e-42	145.2	24.8	*
	u_UniCraF_334	3.80e-34	119.8	20.2	*
	u_PotAlaF_317	9.30e-34	118.5	21.5	*
	u_LamGotF_340	4.60e-33	116.2	9.4	*
	u_UniTumF_325	5.60e-32	112.6	24.0	*
	u_UniDelF_344	2.90e-31	110.3	19.3	*
	h_WesCarF_330	3.30e-31	110.1	22.7	*
	m_MarCraF_334	4.60e-29	103.0	33.8	*
	u_LamTorF_317	2.50e-28	100.6	16.6	*
	u_PygGraF_310	1.10e-27	98.5	8.9	*
	m_MarFalH_322	2.90e-27	97.1	42.9	
	m_CumMonF_319	4.90e-27	96.3	35.0	*
	u_VenEllF_322	5.90e-27	96.1	22.0	*
	u_ArcLanF_342	7.10e-27	95.8	23.0	*
	u_AnoAnaF_353	9.30e-27	95.4	13.6	*
	u_LasComH_332	1.10e-25	91.9	38.0	
	u_SinWooF_343	2.70e-25	90.6	21.5	*
	u_LamLeaF_338	3.40e-25	90.3	20.2	*
	u_UttPenF_329	1.80e-23	84.6	2.7	*
	u_QuaQuaF_339	1.20e-22	81.9	15.4	*
	u_PotLitF_316	8.90e-22	79.0	11.0	*
	u_UniJapF_340	1.30e-21	78.5	9.5	*
	h_EchMenF_311	1.30e-21	78.5	26.4	*
	u_SolCarF_363	1.50e-17	65.0	18.2	*
	u_ToxParH_333	0.041	14.2	4.6	
	u_PotAlaM_435	0.14	12.4	3.4	between *nad5* and *trnH*
	h_WesCarF_518	0.38	11.0	3.4	
	m_MarMrcM_383	0.46	10.7	0.7	inside *16S*, same direction
	u_VenEllF_520	0.48	10.6	5.5	inside *cox3*, opposite direction
	u_LamTorF_541	0.49	10.6	1.5	inside *cox1*, opposite direction
	m_MarMarF_513	0.49	10.6	3.1	inside *cox1*, opposite direction
	h_PriOblF_374	0.64	10.2	4.5	end of *cob*, same direction
	u_UniDelF_517	0.66	10.2	2.1	inside *nad4*, opposite direction
	u_VenEllM_476	0.69	10.1	2.7	overlaps *trnH*, opposite strand
	u_VenEllM_471	0.71	10.1	0.3	inside *nad5*, opposite direction
	u_VenEllF_177	0.86	9.8	1.0	inside *cob*, opposite direction
	u_PygGraM_411	0.89	9.8	3.6	between *trnF* and *nad5*
	y_FosFosF_544	0.96	9.7	1.5	inside *cox3*, opposite direction
	u_PygGraF_283	1.1	9.5	0.8	inside *cox2*, opposite direction
	u_UttImbH_309	1.1	9.5	1.2	inside *cox2*, opposite direction
	i_MutDub_579	1.1	9.4	5.3	inside *cox3*, opposite direction

HMM Profile	Sequence	E-value	Score	Bias	Description
	u_ToxParH_473	1.4	9.1	1.8	inside *nad6*, same direction
	u_AnoAnaM_514	1.4	9.1	0.8	inside *nad4*, opposite direction
	u_UniDelM_452	1.6	8.9	0.8	inside *nad5*, opposite direction
	m_MarDahF_534	1.6	8.9	0.3	inside *cox3*, opposite direction
hmm_FORF	u_LasSubH_326	2	8.6	1.3	inside *nad1*, same direction
	h_EchMenM_445	2	8.6	2.7	overlaps *trnH*, opposite strand
	u_ArcLanF_293	2.2	8.5	1.3	inside *cox2*, same direction
	u_LamTorF_463	2.3	8.4	3.5	overlaps end of *nad6*, same direction
	u_PotAlaF_506	2.3	8.4	25.3	inside *cox3*, opposite direction
	u_QuaQuaM_516	2.6	8.2	0.9	inside *nad4L*, opposite direction
	u_LamGotF_299	2.6	8.2	3.3	inside *cox2*, same direction
	u_UniTumM_506	2.7	8.2	1.3	overlaps 5' of *atp8*, opposite direction
	m_MarMarM_314	2.8	8.1	0.8	inside *cox2*, opposite direction
	u_UniJapM_187	3.2	7.9	3.2	overlaps *cob* and upstream tRNAs, opposite direction
	u_LamTorF_513	3.3	7.9	1.2	between *atp6* and *cox3*, opposite direction
	y_LamEnsF_443	3.3	7.9	4.2	inside *nad1*, same direction
	i_ChaRub_573	3.3	7.9	0.2	inside *cox1*, opposite direction
	u_AnoAnaF_562	3.4	7.9	3.4	inside *cox3*, opposite direction
	u_UniTumM_501	3.6	7.8	4.0	5' of M-*orf*, opposite direction
	y_AnoTra_420	3.8	7.7	3.9	inside *nad5*, same direction
	m_MarMarF_473	4.2	7.5	0.6	between *nad4L* and *atp8*, opposite direction
	u_PygGraF_546	4.4	7.5	0.3	inside *cox1*, opposite direction
	h_WesCarM_523	4.5	7.4	0.7	inside *cox1*, opposite direction
	u_VenEllF_391	4.6	7.4	2.0	inside *cob*, same direction
	u_VenEllF_485	4.7	7.4	2.7	inside *nad4*, opposite direction
	u_PotAlaM_538	4.9	7.3	1.9	overlaps 3' of *cox1*, opposite direction
	h_WesCarM_363	5.2	7.2	0.1	inside *16S*, same direction
	h_WesCarF_324	5.2	7.2	4.7	overlaps *trnS1*, same direction
	m_MarFalH_485	5.3	7.2	3.5	inside *nad4*, opposite direction
	u_PotAlaF_333	5.4	7.2	0.8	inside *12S*, opposite direction
	h_EchMenM_447	5.5	7.2	1.4	in unassigned region between *trnH* and *trnV*
	m_MarDahF_552	5.5	7.1	1.4	inside *cox1*, opposite direction
	u_LasComH_480	5.7	7.1	2.5	inside *nad4*, same direction
	u_ToxParH_391	5.8	7.1	1.7	overlaps *trnL* and *trnN*, same direction
	u_PotAlaM_515	5.9	7.1	1.7	inside *atp6*, opposite direction
	m_MarAurF_519	5.9	7.1	1.8	inside *cox1*, opposite direction
	m_MarCraF_553	5.9	7.1	1.7	inside *cox1*, opposite direction
	u_LasComH_571	6	7.0	0.8	inside *cox1*, opposite direction
	u_LasSubH_415	6	7.0	0.8	inside *cox1*, opposite direction
	h_PriOblF_305	6.1	7.0	1.8	inside *nad2*, same direction
	h_EchMenF_333	6.2	7.0	0.4	inside *12S*, same direction
	h_EchMenM_560	6.4	6.9	0.7	inside *cox1*, opposite direction
	u_UttPenF_554	6.4	6.9	1.0	inside *cox1*, opposite direction
	m_MarMrcM_467	6.5	6.9	4.5	inside *nad4*, opposite direction
	h_WesCarM_463	6.8	6.9	9.7	inside *nad4*, opposite direction
	u_UniCraF_284	7	6.8	3.3	inside *cox2*, same direction
	u_PygGraF_537	7	6.8	1.2	inside *cox1*, opposite direction
	h_CasAmbF_543	7.5	6.7	1.9	inside *cox1*, opposite direction
	u_SinWooF_247	7.5	6.7	0.1	between *trnM* and *nad2*, opposite direction
	u_LamLeaM_380	7.6	6.7	0.3	inside *16S*, same direction
	u_SinWooF_525	7.7	6.7	1.0	inside *nad4*, opposite direction
	m_MarFalH_539	7.8	6.7	2.9	inside *cox1*, opposite direction
	m_MarCraF_379	8.3	6.6	0.0	inside *16S*, same direction
	h_PriOblF_392	8.3	6.6	0.4	inside *nad5*, same direction
	h_PriOblF_306	8.5	6.5	0.7	inside *nad2*, same direction
	u_UniCraF_555	8.9	6.5	2.4	inside *cox1*, opposite direction
	u_UniDelF_580	8.9	6.5	2.4	inside *cox1*, opposite direction
	u_PygGraF_467	8.9	6.5	0.1	inside *nad4*, opposite direction
	u_LamLeaM_529	9.2	6.4	0.9	between *atp6* and *cox3*, opposite direction
	u_ToxParH_486	9.3	6.4	0.5	inside *nad4*, opposite direction
	u_PotLitM_558	9.3	6.4	1.0	inside *cox1*, opposite direction
	m_MarMrcF_435	9.3	6.4	1.1	unassigned region between *trnQ* and *nad5*
	u_UttPenF_339	9.3	6.4	0.2	inside *nad2*, same direction
	u_PygGraM_98	9.4	6.4	0.2	between *nad6* and *nad1*, opposite direction
	u_LamGotM_479	9.6	6.4	0.5	between *nad6* and *nad4*, opposite direction
	u_SinWooM_597	9.8	6.3	0.9	inside *atp6*, opposite direction
	u_PygGraM_453	9.8	6.3	0.1	inside tRNA cluster upstream *nad1*, same direction
hmm_MORF01	u_PygGraM_64	2.20e-126	424.5	48.5	*
	h_EchMenM_55	1.80e-117	395.0	67.2	*
	u_SinWooM_77	6.30e-112	376.6	38.7	*
	u_LamTorM_61	1.60e-110	372.0	54.0	*
	u_UniCraM_62	1.70e-105	355.4	48.4	*
	u_ArcLanM_55	1.30e-104	352.4	48.2	*
	u_UniTumM_61	1.10e-103	349.4	47.1	*
	u_LamGotM_63	2.40e-102	344.9	53.1	*
	u_AnoAnaM_62	7.20e-102	343.3	41.6	*
	u_UniDelM_58	7.20e-101	340.0	37.8	*
	u_UttPenM_56	1.80e-94	318.8	42.6	*
	h_WesCarM_56	1.70e-72	246.1	75.1	*
	u_VenEllM_59	2.80e-67	228.7	53.8	*
	u_SinWooM_66	5.80e-65	221.1	42.9	*, corresponds to M-*orf*b
	u_PotAlaM_63	2.20e-48	166.2	88.1	*

HMM Profile	Sequence	E-value	Score	Bias	Description
	u_SolCarM_58	4.00e-43	148.8	50.6	*
	u_UniJapM_58	3.60e-35	122.4	43.4	*, corresponds to M-*orf*, overlaps upstream *trnD*
	u_LamLeaM_52	6.10e-30	105.1	42.8	*, corresponds to M-*orf*, overlaps upstream *trnD*
	u_PotLitM_53	2.30e-28	99.8	42.2	*, corresponds to M-*orf*, overlaps upstream *atp6*, *atp8* (different frame), *trnD*, and downstream *nad4L* (different frame)
hmm_MORF01	m_MarMarM_78	6.70e-21	75.1	30.5	*
	m_CumMonM_64	2.70e-20	73.1	16.2	*
	m_MarMrcM_71	3.10e-18	66.3	46.8	*
	u_QuaQuaM_67	2.40e-10	40.2	54.9	*
	u_LamLeaF_56	1.8	7.4	3.4	corresponds to *atp8*
	u_UniJapF_210	3.6	6.5	0.6	inside *12S*, opposite direction
	u_LamLeaF_113	3.9	6.3	0.2	inside tRNA cluster upstream *nad1*, opposite direction
	u_SolCarM_371	4	6.3	1.3	inside *12S*, same direction
	u_LamLeaM_46	4.2	6.2	2.6	between *atp8* and *trnD*, same direction
	u_ArcLanM_50	4.8	6.1	0.1	between *trnD* and M-*orf*, same direction
	u_VenEllM_388	4.9	6.0	0.0	between *trnY* and *12S*, same direction
	u_LamTorF_137	5.3	5.9	0.7	inside *nad5*, same direction
	m_MarFalH_73	5.9	5.7	1.6	inside *nad4*, same direction
	y_AnoEloF_278	6	5.7	5.5	in unassigned region between *trnA* and *trnH*
	h_EchMenF_352	6.3	5.6	0.1	inside *16S*, same direction
	h_CasAmbF_553	7.3	5.4	0.2	inside *cox1*, opposite direction
	m_CumMonF_202	7.4	5.4	1.1	inside *16S*, opposite direction
	u_LamGotM_66	9.5	5.1	1.4	inside *nad4L*, same direction
	u_QuaQuaM_226	9.6	5.0	0.7	inside *16S*, opposite direction
	u_VenEllM_90	9.8	5.0	0.7	inside *nad4L*, same direction
hmm_MORF02	m_MarMrcM_62	3.10e-95	319.6	45.0	*
	m_CumMonM_56	6.30e-87	292.2	33.7	*
	m_MarMarM_70	1.90e-84	284.1	40.5	*
	m_MarMrcM_71	3.80e-14	52.4	11.9	
	m_CumMonM_64	8.20e-13	48.0	16.0	
	m_MarMarM_78	5.90e-09	35.3	15.9	
	u_UniTumF_20	0.22	10.3	0.8	inside *cox1*, same direction
	i_MutDub_143	0.25	10.1	2.1	inside *nad5*, same direction
	u_PotAlaM_223	0.45	9.3	2.3	inside *12S*, opposite direction
	y_AnoTra_350	0.79	8.5	0.4	between *12S* and *trnK*, same direction
	m_MarMarM_275	1.7	7.4	0.1	inside *nad2*, opposite direction
	m_MarMrcM_98	2	7.2	1.7	inside *nad6*, opposite direction
	u_PygGraM_154	2	7.1	0.6	inside *cob*, opposite direction
	u_LamLeaF_21	2.1	7.1	0.9	inside *cox1*, same direction
	h_WesCarM_211	2.2	7.0	0.5	inside *16S*, opposite direction
	u_PotAlaM_81	2.6	6.8	0.9	inside *nad4*, same direction
	u_UttPenM_85	2.9	6.6	0.2	inside *nad4*, same direction
	u_UniTumM_16	3	6.6	6.8	inside *cox1*, same direction
	u_VenEllM_281	3.2	6.5	2.8	5' of *nad2*, opposite direction
	u_UttPenM_243	3.5	6.3	5.7	inside *nad2*, opposite direction
	y_LamEnsF_47	3.8	6.2	2.7	inside *atp6*, same direction
	u_QuaQuaM_97	3.8	6.2	1.6	inside *nad6*, opposite direction
	u_UniDelM_249	4.7	5.9	0.8	inside *nad2*, opposite direction
	u_LasComH_253	5	5.8	0.4	overlaps with 5' end of *nad2*, same direction
	m_MarDahF_358	5.3	5.8	0.0	inside *12S*, same direction
	m_MarFalH_350	5.3	5.8	0.0	inside *12S*, same direction
	m_MarMarF_326	5.3	5.8	0.0	inside *12S*, same direction
	h_EchMenM_283	5.3	5.8	1.8	5' of *nad3*, same direction
	u_SolCarM_38	5.6	5.7	1.5	inside *atp6*, same direction
	u_QuaQuaF_125	5.8	5.6	1.5	in unassigned region between *trnQ* and *nad5*
	m_MarAurF_342	5.9	5.6	0.0	inside *12S*, same direction
	u_LamGotM_14	6	5.6	2.9	inside *cox1*, same direction
	u_LamTorM_13	6	5.6	0.3	inside *cox1*, same direction
	u_UniJapF_61	6	5.6	0.2	inside *nad4*, same direction
	m_MarCraF_61	6.7	5.4	0.5	inside *nad4*, same direction
	u_UniDelF_424	7.4	5.3	0.0	inside *cob*, same direction
	h_CasAmbF_260	7.5	5.3	0.7	between *trnA* and *trnH*
	h_EchMenM_190	7.7	5.2	0.1	between *cob* 5' and tRNAs, opposite direction
	m_MarMrcF_346	8.1	5.1	0.0	inside *12S*, same direction
	m_CumMonF_234	8.1	5.1	0.3	inside *12S*, same direction
	y_LamEnsF_37	8.9	5.0	2.7	inside *cox3*, same direction
	u_AnoAnaM_588	8.9	5.0	0.3	inside *cox1*, opposite direction
	u_LamLeaF_60	9.2	5.0	0.2	inside *nad4L*, same direction
	u_LamTorF_205	9.3	4.9	0.3	inside *16S*, opposite direction
	u_PotAlaM_262	9.4	4.9	2.0	5' of *nad2*, opposite direction
	u_UniCraF_94	9.8	4.9	1.1	inside *nad6*, opposite direction
	u_SolCarF_619	9.9	4.9	0.0	between *cox1* and *cox2*, opposite direction
	u_UttImbH_114	9.9	4.9	0.1	inside *nad1*, opposite direction
hmm_MORF12	h_EchMenM_55	1.40e-123	415.3	68.3	*
	u_PygGraM_64	4.60e-120	403.7	46.9	*
	u_SinWooM_77	6.60e-106	356.8	39.4	*
	u_LamTorM_61	8.70e-106	356.4	53.5	*
	u_UniCraM_62	1.60e-102	345.5	47.4	*
	u_ArcLanM_55	1.20e-99	336.1	47.5	*
	u_UniTumM_61	5.60e-99	333.8	47.4	*
	u_AnoAnaM_62	2.30e-97	328.4	41.5	*
	u_LamGotM_63	2.90e-97	328.1	52.8	*

HMM Profile	Sequence	E-value	Score	Bias	Description
	u_UniDelM_58	1.50e-96	325.7	38.2	*
	u_UttPenM_56	1.70e-90	305.7	41.4	*
	h_WesCarM_56	6.80e-79	267.3	74.5	*
	u_VenEllM_59	5.90e-63	214.4	54.2	*
	u_SinWooM_66	6.40e-61	207.7	42.8	*, corresponds to M-*orf*b
hmm_MORF12	u_PotAlaM_63	1.50e-50	173.4	68.0	*
	u_SolCarM_58	1.00e-40	140.7	48.1	*
	u_UniJapM_58	2.30e-33	116.4	41.1	*, corresponds to M-*orf*, overlaps upstream *trnD*
	m_MarMrcM_62	7.30e-29	101.5	25.6	*
	u_LamLeaM_52	2.00e-28	100.0	41.1	*, corresponds to M-*orf*, overlaps upstream *trnD*
	u_PotLitM_53	1.80e-26	93.6	5.8	*, corresponds to M-*orf*, overlaps upstream *atp6*, *atp8* (different frame), *trnD*, and downstream *nad4L* (different frame)
	m_CumMonM_56	1.60e-21	77.2	16.0	*
	m_MarMarM_70	6.10e-20	71.9	18.2	*
	m_CumMonM_64	4.70e-18	65.7	15.9	*
	m_MarMarM_78	1.20e-17	64.3	7.6	*
	m_MarMrcM_71	5.70e-16	58.8	47.9	*
	u_QuaQuaM_67	1.30e-07	31.1	55.6	*
	u_SolCarM_199	0.73	8.7	1.0	overlaps *trnL*, opposite direction
	m_MarCraF_88	1.5	7.7	0.6	inside *nad6*, opposite direction
	h_EchMenF_50	1.6	7.6	8.0	between *trnD* and *atp8*, same direction
	u_LamLeaF_113	1.7	7.5	0.1	between *trnL* and *trnV*, opposite direction
	y_AnoEloF_278	1.8	7.4	5.8	in unassigned region between *trnA* and *trnH*
	u_ToxParH_222	2.1	7.2	1.5	between *16S* and *trnY*, opposite direction
	u_ArcLanM_50	3.1	6.7	0.4	between *trnD* and M-*orf*, same direction
	u_LamLeaF_319	4.2	6.2	1.8	between *nad2* and *trnM*, opposite direction
	u_LamGotM_66	4.2	6.2	1.4	inside *nad4L*, same direction
	u_LamLeaF_121	4.8	6.0	1.9	in unassigned region between *trnQ* and *nad5*
	m_MarCraF_149	5.9	5.7	0.1	inside *nad5*, same direction
	u_ArcLanM_214	6.4	5.6	0.3	inside *16S*, opposite direction
	u_ArcLanF_55	6.7	5.5	8.1	between *trnD* and *atp8*, in frame with *atp8*, whole length of *atp8*
	u_UniCraM_151	7.2	5.4	2.7	inside *nad5*, same direction
	u_UniCraF_144	7.9	5.3	0.9	inside *nad5*, same direction
	m_CumMonM_55	8.1	5.3	6.8	end of M-*orf*2, same direction
	u_LasComH_263	9.3	5.1	4.5	overlaps 5' of H-*orf*, opposite direction
	u_ArcLanF_196	9.4	5.0	0.8	inside *16S*, opposite direction

Table S4

Summary of the phmmer analysis. The translated protein sequences from the two *Anodontites trapesialis* ORFans were used as input sequences (‘Query Name’). For each sequence identified (‘Sequence’) in the set of 35,032 ORFs extracted from all considered mtDNAs with getorf, are shown the full sequence statistics (‘E-value’, ‘Score’, ‘Bias’) taken from the output produced by phmmer, with a short description of the hits. Hits below the inclusion threshold of phmmer are separated by a dotted line in the table.

Species acronyms for the new mt genomes sequenced in this study appearing in the column ‘Target Name’: ChaRub for *Chambardia rubens*, AnoElo for *Anodontites elongata*, FosFos for *Fossula fossiculifera*, LamEns for *Lamproscapha ensiformis*, MonPar for *Monocondylaea parchappii*, CasAmb for *Castalia ambigua*, DipSua for *Diplodon suavidicus*, PriObl for *Prisodon obliquus*, WesCar for *Westralunio carteri*, MarCra for *Pseudunio auricularius* (formerly *Margaritifera auricularia*, used during data elaboration). Other acronyms and their respective species names are enlisted in Table S1.

Query Name	Sequence	E-value	Score	Bias	Description
AtraUR219	y_AnoTra_77	2.00e-40	136.3	5.1	corresponds to *AtraUR219*, overlaps upstream *trnD*
	y_AnoTra_61	0.028	16.0	0.1	overlaps end of *atp8*, same direction (different frame)
	u_PygGraM_81	1.4	10.6	0.2	inside *nad4*, same direction
	u_AnoAnaM_207	2.7	9.7	0.8	inside *16S*, opposite direction
	m_MarDahF_92	2.8	9.7	0.4	between *nad6* and *nad1*, opposite direction
	u_UttPenM_9	3.5	9.4	2.7	inside *cox1*, same direction
	u_SinWooF_191	3.6	9.4	0.0	inside *16S*, opposite direction
	u_UniTumM_448	3.8	9.3	0.2	between *trnL* and *nad1*, same direction
	u_UniCraM_36	4.1	9.2	1.0	between *cox3* and *atp6*, same direction
	u_SinWooF_148	4.4	9.1	0.8	inside *nad5*, same direction
	u_ArcLanM_283	4.9	8.9	2.3	inside *nad3*, same direction
	m_MarFalH_53	5.3	8.8	0.3	corresponds to *atp8*
	h_WesCarF_268	5.4	8.8	3.0	unassigned region between *trnS1* and *trnS2* (upstream F-*orf*)
	u_UniDelM_32	5.7	8.7	2.3	between *cox3* and *atp6*, same direction
	u_AnoAnaF_446	6.3	8.6	0.6	between *cob* and *trnF*, same direction
	y_MonParF_58	7.1	8.4	0.4	corresponds to *atp8*
	m_MarMrcF_341	7.8	8.3	0.4	inside *12S*, same direction
	u_LamTorF_402	8.7	8.1	0.5	inside *cob*, same direction
	u_AnoAnaM_10	9	8.1	1.2	inside *cox1*, same direction
	h_WesCarM_23	9.2	8.1	4.4	inside *cox3*, same direction
	u_VenEllF_460	9.3	8.1	0.1	inside *nad1*, same direction
	u_QuaQuaM_215	9.3	8.0	0.4	inside *16S*, opposite direction
	u_UniJapF_454	9.7	8.0	0.3	unassigned region upstream *nad5*, probable sequencing gap inside
	u_SinWooF_115	9.7	8.0	0.7	overlaps *trnV* into upstream unassigned region, opposite direction
	u_UniJapM_213	9.8	8.0	1.3	inside *16S*, opposite direction
AtraUR2218	y_AnoTra_68	8.60e-11	40.0	5.3	corresponds to *AtraUR2218*
	h_WesCarF_62	0.085	14.0	2.1	inside *nad4*, same direction
	m_MarMrcF_235	0.17	13.2	0.5	between *12S* and *trnR*, opposite direction
	u_PotAlaF_39	1.2	10.7	2.9	inside *atp6*, same direction
	u_PotLitF_127	1.8	10.2	5.4	inside *nad5*, same direction
	u_ArcLanF_85	1.9	10.1	0.4	inside *nad6*, opposite direction
	u_PygGraM_80	2.6	9.8	0.3	inside *nad4*, same direction
	h_WesCarM_221	2.6	9.8	1.4	between *16S* and *trnY*, opposite direction
	u_UniJapF_237	2.8	9.7	0.2	inside *12S*, opposite direction
	m_MarFalH_130	2.8	9.7	1.1	inside *nad5*, same direction
	u_QuaQuaF_50	2.8	9.6	6.4	overlaps *trnD* and *atp8*, same direction
	y_LamEnsF_260	3	9.6	0.5	inside *nad2*, opposite direction
	u_AnoAnaF_47	3.6	9.4	0.3	inside *atp6*, same direction
	u_LamLeaM_70	4.6	9.0	4.9	inside *nad4*, same direction
	y_MonParF_286	4.6	9.0	1.2	overlaps 3' end of *trnH*, same direction
	m_MarFalH_44	4.8	9.0	1.5	inside *atp6*, same direction
	m_MarDahF_247	4.9	9.0	0.1	inside *nad2*, opposite direction
	h_WesCarF_38	5	8.9	0.4	inside *atp6*, same direction
	m_CumMonF_279	5.1	8.9	2.6	between *nad3* and *cox2*, same direction
	y_FosFosF_10	5.4	8.8	6.0	inside *cox1*, same direction
	h_WesCarF_75	5.7	8.8	0.3	inside *nad4*, same direction
	y_AnoEloF_203	5.8	8.7	4.9	inside *16S*, opposite direction
	u_SinWooM_92	5.9	8.7	2.1	inside *nad4*, same direction
	u_ArcLanM_21	6.3	8.6	0.5	overlaps 5' of *cox3*, same direction
	y_AnoTra_12	6.4	8.6	3.4	inside *cox1*, same direction
	u_PygGraF_139	6.6	8.6	3.3	unassigned region between *nad5* and *trnF*
	m_MarMrcF_220	6.6	8.6	1.5	inside *12S*, opposite direction
	u_VenEllM_112	6.6	8.6	0.1	inside *nad1*, opposite direction
	u_UniJapM_282	6.6	8.6	0.1	inside *cox2*, same direction

Query Name	Sequence	E-value	Score	Bias	Description
AtraUR2218	m_MarMrcF_139	6.8	8.5	2.1	inside *nad5*, same direction
	m_CumMonF_73	7	8.5	0.8	inside *nad4*, same direction
	u_VenEllM_271	7.1	8.5	0.7	inside *nad2*, opposite direction
	m_CumMonF_219	7.2	8.5	0.6	inside *12S*, opposite direction
	u_UniDelM_74	7.5	8.4	1.1	inside *nad4*, same direction
	h_DipSuaF_120	7.6	8.4	0.4	inside *nad6*, opposite direction
	m_MarCraF_18	7.9	8.4	1.7	inside *cox1*, same direction
	u_SolCarM_149	8.1	8.3	0.4	inside *nad5*, same direction
	y_AnoTra_91	8.3	8.3	0.5	inside *nad4*, same direction
	y_LamEnsF_44	8.7	8.2	1.1	inside *atp6*, same direction
	h_EchMenM_211	8.9	8.2	0.7	inside 16S, opposite direction
	u_UniDelM_163	9	8.2	0.3	inside cob, opposite direction
	m_MarMarM_75	9	8.2	0.5	overlaps *trnD* and M-*orf*1, same direction
	u_LamTorF_75	9.3	8.2	0.9	inside *nad4*, same direction
	u_UniTumM_139	9.5	8.1	1.8	inside *nad5*, same direction
	u_LamLeaF_181	9.8	8.1	0.4	between *trnL* and *16S*, opposite direction
	u_UttImbH_78	9.8	8.1	0.2	inside *nad4*, same direction
	u_UniJapF_180	9.9	8.1	1.0	inside *cob*, opposite direction
