# Supplementary material for: Variability of mitochondrial ORFans hints at possible differences in the system of doubly uniparental inheritance of mitochondria among families of freshwater mussels (Bivalvia: Unionida)
Source: BMC Evol Biol. 2019 Dec 19;19:229. doi: 10.1186/s12862-019-1554-5 (PMC6923999; doi:10.1186/s12862-019-1554-5)
Supplement: Supplementary file 2 — Additional file 2. List of mitochondrial ORFan gene sequences of freshwater mussels whose translated protein products were used in this study. [file 12862_2019_1554_MOESM2_ESM.pdf]

ADDITIONAL FILE 2

Nucleotide sequences in fasta format of the ORFan genes F-*orf*, H-*orf*, M-*orf*, and the two ORFans from *Anodontites trapesialis*, whose translated amino acid sequences were analyzed in this study. Each sequence name (apart from *A. trapesialis* ORFans, which follow the nomenclature of past literature for uniformity) is composed by: a letter indicating the family of the species (h, Hyriidae; m, Margaritiferidae; u, Unionidae; y, Mulleriidae = Mycetopodidae), the acronym of the species (see Additional file 1), and the name of the ORFan. All three Margaritiferidae species have two M-*orf* genes (numbered 1 and 2, following past literature), and *Sinanodonta woodiana* (Unionidae) has a species-specific duplication of the M-*orf* (the two copies are here named a and b).
The species acronyms appearing here GibCra, PseAur, PseMrc, AcuTor, LanLan, ProJap, and Schsp stand for the updated and more correct species names *Gibbosula crassa*, *Pseudunio auricularius*, *Pseudunio marocanus*, *Aculamprotula tortuosa*, *Lanceolaria lanceolata*, *Pronodularia japanensis*, and *Schistodesmus sp*. (formerly *Margaritifera crassa*, *Margaritifera auricularia*, *Margaritifera marocana*, *Lamprotula tortuosa*, *Arconaia lanceolata*, *Unio japanensis*, and *Lamprotula gottschei* during data elaboration; see also Additional file 1).

F-*orf* (25 sequences)

```
>h_EchMenF_Forf
ATCAAAAAGCCCAGACTAAGAAGCCCAAAAAACCCTATAATTATTATAGCCGCCCTGCTTACCCTCCTGCTTATCACCATTATTTGCTATACTTAATAAGACATGGCCAAGATTCCACAACT
AGGTTAACCATTACCAGCATAGACATCACAGATATAACCTCAGAAAACCTACAAACAAAAGGTACTAACCCACAACAAAATGACACACCAACCGGACACACCCCACACAAAAGCAAAGCTCAC
ACCAACCTAAACACAAAGTAG
>h_WesCarF_Forf
ATCAAAGTCAAGACTTTTAACACACCTACCAAATACATCATTACATCACTTTTTATTCTAGGTATAGCGTACCTAGCCCTACGCACACACACAAGCCCTGCTGATATGTTTCCAACCGATGCT
TCACAAATCCCAACAACTAACATAGCCACAGAACCAATCACAAACGAAGAAACAAACATAGTCCAACCAATACAAAGCCTACCAGCAAACATGGCCAATCTTAAGCAAGAAAACACTCAAACA
GACCACAACTTAACTAGCCACAACCCACAAAAAAGTAAAGCCAACACCAACCTCACATCAAAATAG
>m_CumMonF_Forf
ATTGCCATCATAACCTTAATTATCTTGATCCCACTCTCGTATTTGCCCCTAATTTGATCAAACACCGACAACCTAAAAACGGCCAACAACCTAAAGATAAAACCAATCGCTCATGACCTCAAA
CCCTCAAAACATCCCACTTCAAATATCACCAAACCACAACCAAACGATACCCAAACCTCAAACGAACATAGACCCAACACCTACAAACCAAAAAAGAGTAAAGCATCAACAAACCTCACCAAT
GACAAACCAAACGCTACAAAAGAACCATAA
>m_GibCraF_Forf
ATTATTAAAAAAACACCAACTATTAAACACCTCCTGGATTTTTTCTCAAACCCCAACTCACCTAAAATCTTAATTTCTATCGCTACCCTACTACTACTACTCTTACTCTCACTACCCCCAATA
GAAACCACAAATGACCTAGAACCAGTAAAACCAACAAAATCACTAACACTAGACTCAACTCAACCAAAGACCTGTAATACCCAAACTAAAAGTGACTCTCATCCAGATACCTACAGACCAAAA
AAAAGTAAAGCATCCACAAGCCTACCCCTAGACTCTAAACCAAGCACACCTAAATCCCCATAA
>m_MarDahF_Forf
ATCTGAAAAACACCAATCATTCTTCAACACCTACGCAATTACTTTTCAAACTCCAACCCGCTCAGAATACTAACTGCTATTATCATTTTAGCATTATTAACAAACCTACTCCTTAATAGAAGA
AGTATTAACGACCTAGATCCAGTAAAACCGCTAACCCTAAACACCAACGAACCACAACCCAACTGTACCAAAACCACAAACAGACACGAACCCAGCGCCTACGAACCAAAGAAAAGCAAAGCA
TCCACAAACCTTGCTACCAATAATAAACCAAACACGCCAGAGTAA
>m_MarMarF_Forf
ATTTGGCATTGGTTAACGAACCTCTTGCCTATCCGAAAAACACCGAGCATCTTTCAACGCCTCCGCAATTACCCATTAAAACATAAACCCCTTTGAACGCTAATCACTGTTAGCACCCTAGCA
TTAATAACAATGATACTTCTCACCAGGGCAAGCGTCAATGATCTAACACCCATAAACCCCACAAAACCGCTAACCATAAACACCACCGAACTACAACCCAGCTGTGTTATAACCACAAGTAAA
CAAGAACCCAGCGCCTACGAACCAAAAAAGAGCAAAGCGTCCACAGATCTTGTTACCGATAAAGAACCATCTCCTCAAGATAAATAG
>m_PseAurF_Forf
GTGCTAGAGCCACAACAAAGATACTGACAAACCAATCACCAAACTATAAATTTGGTACAGGTCAATGACCTCTTTTTCATCAAAAAAACACCAATCATTCAACGCCTCCATGACTACCTCTTA
AACCTAAATCCTCTAAGAATCACAATCACTATGACTATATTAATGCTACTGACAAACCTATTAATTGAGCCGATCAGCGTAAACGACCCACAATCGATTGAAACCCCAAAACCACTTGCCTTA
AATACCGCCGAACCACACCTTAACGACCCCAAAATCACAGATGAACACGAAACCGGCACTTACGCACCAAAGGAAAGTAAAGCGTCCACAAACCTCACCACCGAAGTTAAAGCGTCCACAAAC
CTCACCCCCGAAGTTAAACCAAACACGCCAAAACAGACCTAG
>m_PseMrcF_Forf
ATTACTAACCAAACTTGAAATTTGGTACAGGTTAATAACCTACCTGTCATCTGAAAAACACGAATTATCCAACACCTCCGCAATCATTCCCATAAATTAAACCCGCTAAAAATTATAATTGCT
ATAGCTATCCTAACACTACTGACAAACCTTTTAACCAAACCAATCGCAAACAACCTGAAAACAATCAAACCCACAAAATCGCACCTCAATGACCTGGGAGTAACAGAGGAACACGAAATAGGC
ACCAACAAACCAAAGAAGAGAAAAGCGTCCACAAACCTCACTCCAGCGACTAAGCCTAATACACCAGAACAAACCTAA
>u_AcuTorF_Forf
ATATTCAAACAAAAACTAATAAGCCTACCATTCTTGAGTCTATTTTTCTCTACTATCATCCTTTGCTTAATCCTAAACACTTCTCCAACCTCTTTCCTCAATGAACCAAAACTTAACGATCAA
ACTCTATGCTCAATGGATCTAAACAACCCTGCATTACAACCGAACCCTGGCCCAGAAGATCACCCAATTATCTCGAGTTCTGCTAAGACAGACCTTACCAAGCCACACCTAGACGCCAAATAA
>u_AnoAnaF_Forf
ATTTCAAATAAATCTTTAATAAAAACTATTTTATTAATCTTATCTATAATTATTCTAACTATCCTTTTAGCCCAAGCTATTCAAATATTCCTCACATCAAGTGAACTCTGAAAGATCGACCAA
ATTTTATGCTCAATAGACTTAGGTAGCACCATTCCTCAACCAGAAACCAGTGATCATCCAGTGATCCCAAGACTTGCTAGAACAGATCTTACTAAACCAGTAATTAAACCTTAA
>u_LamLeaF_Forf
ATCCAAAAGTCCACTATAAATCTAAAATTACTCGTTTTAATTAGACTTTGCCTGCTACTTCTCTTTGAAATTATATACTATGGTATACCAACCAGCCTAATAGTCTCCGCCACAGACCCACTA
ACATTATCTGATCAACCGTCAACCATCAACTCAACCATTAACGAGCCCAATAACCTTACGACCCCCACAACCCAAGAAAATCACACTGTAATGCCCAGCCAAGCTCACACAGACATTACCAAA
ACATAA
>u_LanLanF_Forf
ATTTTTAAACAAAAACTGCCAAACCTACTGCTTTTAAGTTTATTTATCTCTCTTATTATTTTTTGTTTAGCTCCAACTACTTCGACCCTTATTCTCCCCAATGAGCCAAGGCCCACTAACAAA
ATTCTATGCTCAACAAACCTAGAAAACCCAGCCCCACAACCTGATGACACAGACCATCCAATTATTGCCAGGTCCGCCACAACAAACTTAATTAAACCAAATCAAACAAACACCAAATAG
>u_PotAlaF_Forf
ATCCACCAAGTTTTGGTCATTAAAATAAAAACCTGGCTTAAAAACATACCCCATCACAAAAAAACACAAAAAGCAATTATTATCTTAATCATCAGACTCCCCCTAATAATCCTCCTACCAAAT
CTCTTCCACGAAATACCAGAAAAATTCCTACACACTAATCTCTCGCTGACCGACAATCCACCAAAAGAAAACCAACCAATCAACACACTCCCAACCCCAACAGGTTGCCACCCCCTTAAAAAT
AGCCCCGCTTCTACCAACATCTCCAACAAAGTGTAA
>u_PotLitF_Forf
ATCCAAAAAATTTATCACTAACCTTAAACTCCCAATCCTATTGGGACTCTGTCTCATTCTTACCTGCACAGCCTTATACCACGGTCTACCCGTCAACTTCATAGTATCAGCCATTGATCCACCA
ATACCTACTCTGCCGACACTTACCAACCAAACAACCCCCACAACCACAGGAAATCACGCTGTAATCCCAAGCAAAGCCCAAACAGACATTACCGAGGCTTAG
>u_ProJapF_Forf
ATTTTACTGGGGCTTTGCCTCCTTTTACTTTGTGGAATCCTATACCACGGCATGCCCGCCAACTCAACAGTATCAGCCACAGACCCCCTACCAACCCCCGACTGAAGCTTAGATGAGACTGCC
CACACCACAACCCCTACAGCCCCAAGAGACCACGCTGTAATGCCTAGCCAGGGAAGTACAGACATCACCGAAGCTTAA
>u_PygGraF_Forf
ATAAGCTTAGAAATAAGCAAAGTAATCTTAAAACCCTCATCTAAACTCTTTCTCTTAATGCTCTCTATTTTTACTGTTAGTTTTTTTATTACTAAGGCTGCCCAAACATTTTCTCTATCAGAT
CACTTTTGGTTAATAGACCAAATTCTATGTTCTATAGAATTAGATGACGTTTCTACTCAAATCAGAGCTGATGACCATCCAGTACTTCCAAGGAAAGCCAGAACAGATTTAACTAAACCAAAT
ACCAGGCTTTAA
>u_QuaQuaF_Forf
ATAAACAAGTTTCGTAACAAAACAACTTGAGATTTAATCATTATTGTCGCGATTAGCCTCCTTATGCTGGTCTTATTCCCTAATCTATTAACAATAGCACCTGAAAGAATCAATCAAATTAAA
CCATCATTAACAGACAACCCACTAGATAACAATCAATTGCCTAATACAACACCAACTGACACAGGTACTCACCCAGTTAACAGAAGCCCAGCTTCTACCGACATTTCCGACAAAAAATAA
```

>u_SchspF_Forf
ATCACAAAATTAAAATTACCAAAATGACTATCATTTAGCTTATTTATCTCATTTATTATCCTTTGCCTAATTCCTACTATTCCTTTACTTTTCCTTTCAGACGAGCCAATACTTACTAACCAA
GCATTATACTCAATAGGCCTAAATGACCAAACTCCACAACCTAAAGATGACGATCATCCTGTTATCGCCAGACCTGGCAACACAGACCTAACCAAACCTCAAGTCAAACCATAA
>u_SinWooF_Forf
ATAGTAATTTTAAATAAAGAAAAAATAAAAGCATTTACATTAACCATCACTATTTCTCTTATTATTATAATCTTTCCTCAGATTTCTCTAATATTTTTTACCACAAATAAAGACTACACTACT
GAACAAATCTTGTGTTCATTAGATTTAAATACCACCCTTTCTAAACCCCAATCCGGGGACCACCCTATTATCTCCAGACCTGCTACAACAGACTTAACTACCTCAAACCCTCCCTTATCAAGC
AAGAAAACCCCGTAA
>u_SolCarF_Forf
ATACACCCCAAAATAACCAACTTCCTAGCAATCAGCCTCGCGCTAACTTTTATAACCCTACTGTATAGACCCTGACTCACTCAAACAACATGGGCCATAGACCCTCCGCCAGCCACTTCTACA
GAAATCCATAACCCATCACCAAACGGTAGCGGGGACACAACAATCCCCTCAAATCCAGGAAATTATCCAGTGATCGCCAGACAAAAACACACAAACATCACCCAACCCACAACATCCCAACAA
GCCATAAACCCATAA
>u_UniCraF_Forf
ATCCTTAAACAAAAACTACAAAAATTATTATCCGTTATTTTATTTTTTTTACTTGTTATTCTATGCCTAACTTTAAGTATTCCACTATTCTTTCTTTCAAACGAACCAAAACTTACCAATCAA
ACACTATGCTCAATAGACCTAAACAATCAACCTTTACAACCTAAAGCAGACAACCATCCTATTACCGCCAGATCTGGCAATACAGATCTAACCAAATCAAACCAAACACCAAGTACCAGAACT
AAGTAA
>u_UniDelF_Forf
ATCTCTAATCAAAATCTTCAAAAGCTACTATCCACACTTACGNTTTTTTTCTTAGCTATCCTATGCCTAGCCTTAAGTATTCCCCTATTTATTCTTTCAAACGAACCAAATCTTACTAATCAA
ACACTATGCTCAATAGACCTAAATAATCAACCTCCACAACCCAAAACAGATAGTTACCCTATTACTGCCAGATCAGGGAATACAGACTTGACCAAACCAAACCAAGCATCCAGCGTCAGAAAA
TAA
>u_UniTumF_Forf
ATACAAGTAATCCTTAAACAAAAACTCCTAAACCTATTTTCTATTATTTTGTTAATCTTATTTACCACCCTCTGCTTACTCTTAAGTATTCCCCTATTCTTTCTCTCAAACGAACCTAAACTC
ACCAACCAAACCCTATGCTCAATAAATTTAAGTAACGAATCTTTACAACCTAAAATCGAAGACCATCCAATTACCGCTAGGCCCGGAAACACAGATTTAACTAAATCAACTCAAACATCCAAT
AATAAAACCAAACCTTAA
>u_UttPenF_Forf
ATTCTAAAACTCCCATTTAAACTATACCTTCTAGCCTCACTTATTTTTACTCGCTTTTTTTTCCTTTCTGGCTCGGACCACTTCTTAACCATAAGCCAAGTTTTACACTCTATAGATCTAAAT
GACGCTTCTTCCCAGGCTAGAACCGGAGACCATCCTATTATCCCTAGAAAAGCCAGAACAGATTTAACTAAACCTAATACTAGGGCCTAA
>u_VenEllF_Forf
TTGGTTATAAAAATAAAAACCCAAATTATAAACTTACTAAATAACAAAATAGTACAAAAACTAATTATTATTTTCACCACTGGACTTTTCCTAATAATCATTTTACCAAGCCCCTTCTTACTA
GTATCAACAAAAATCACTTATCCCGAACTGTCACTAACCGATAATCCACCAGAAAAAAATCAACCAACTAGTACATCTACAGCTTCCACCGGATCTTATCCTATCAAAAATAGTCCTGCTTCA
ACCAACATCTCTGACAAAACATAA

H-*orf* (5 sequences)

>m_MarFalH_Horf
ATAAAGGCACCAATAAATTACCTGCCAAACTTTAAAAATTGGCGTAGGTTGACAAACCCCTTCTTTATTTGAAAAAAGATAAACACCCAACCCCTACGCAATTACCCCTCAAAATTTAACCCA
CTAAAAGTGTTAACTGCCATCACTATTCTAGCGCTCTTAACAAGTATACTTTTATTTGCATCAAATACTAATGATTTAAAACCCATCAAACCCACAAAACCACTAACCCTAAACACCACCAAT
CCACAACCCAACTGTACCCAAACCACCACCAATCCACAACCCAACTGTACCCAAACCACCGACAGACACGAACCAAAGAAAAGTAAAGCATCTACCAACCTTACTACTAACGACAAACCAAAA
CACACCAAATAA
>u_LasComH_Horf
ATCATAAAATCCGCACTAAAACTCCTTTTATCAATCTTATCTATTTTCGCTATTGCTTTTTTTTTAATCAAAATTATTGAAGTACTCTTTGTATCAGATGAATTTTGAATACTAAACCAAATT
TTATATTCTGTAGAACTAGATAACGTCTCTTCCCAACCCATAACTGGCAACCATAGCGACCATCCAGTCATCTCAAGACCAGCTAGAACCGATGTCTCTTCCCAACCCATAAATGGCGACCAT
AGCGACAATCCCGTCATCTCAAGACCAGCTAGAACCGATGTCTCTTCCCAACCCATAAATGGCGACCATAACGACCATCCGGTCATCTCAAGACCAGCTAGAACCGATGTCTCTTCCCAACCC
ATAAATGGCGACCATAGCGACCATCCAGTCATCTCAAGACCAGCTAGAACCGATGTCTCTTCCCAACCCATAAATGGCGACCATAACGACCATCCAGTCATCTCAAGACCAGCTAGAACCGAT
GTAACTAAACCAAGTGATAAGCCTTAA
>u_LasSubH_Horf
ATGATAAAGTCCGCTTTAAAACTCCTTTTATTTATTTTATTTAATTTCGCTATTGCTTTTATTTTAATTGAAATTATTCAAGTACTCTTTGAATCAGATGAGTTTTGAATAGTAAACCAAGTT
GTATATTCTATAGAACCAGATAACGCTTTTTCTCAACCCAAAAATGGCGACCATCCAGTTATCGCAAGACCAGCTAGAACCGATGTAACTCAACCCAGAAATGGCAATTATTCAGTTGTCGCA
AGACCAGCTATAACCGATTTAACTCAACTCGGAAATGGCGACCATCCAGTGGTCGCAAGACCAGCTAGAACCGATATAACTCAACTCGGAAGTGGCGACCATCCAGTTATCGCAAGACCAGCT
AGAACCGATGTAACTCAACCCAGAAATGGCAATTATTCAGTTGTCGCAAGACCAGCTAGAACCGATTTAACTCAACCCAGAAATGGCAATTATTCAGTTGTCGCAAGACCAGCTAGAACCGAT
TTAACTCAACCCAGAAATGGCGACCATCCAGTTATCGCAAGACCAGCTAGAACCGATGTAACTCAACCAAGTGCTAAGCCCTAA
>u_ToxParH_Horf
ATCAAACCAAAACACCAACCAAAACAAACACCCAGAGGCCTTCACCCACTTAAAAACAGCTCTGCTTCCACCAGCATCTCTAACAAAGTATTAAATGAAATCTCCACACAAATTCCTCTTTTT
ATTCCTAATGGTTACAAGAACATGAATAGTACTATCCTCCTCCAACTGGCTAACAACCTGAATAGGCCTAGAAATCAACATATTAGGATTTATCCCACTAATATACCTTAA
>u_UttImbH_Horf
ATGACTCATAACAAGTCTAACAACAGGAATATACGCAAAACAATTATAATTTTCTTATGTAAATTATATTGCTTGATTTCTTTTACCTCTATTATCTCCATTATACTAATTGTTGCTTGTTTC
CTCCCCTCTTTGTTTCTTTATAGCTTAGATTACTTTTGGCTTCTCTTAGACCAAGTTTGGATTCAAAATCTACACCTTATAGAAGTATATGCTATTTCTCCTCAGACTAGACTTGAGACTATA
GCTGAGACTATAGCTGAGACTATAGCTGAGACTATAGCTGAGACTAGAGCTCAGACTAGACCTGAGACTGTAGCTGAGACTGTAGCTGAAACTAGAGCTCAAACTAGAGCTCAAACTAGAGCT
CAAACTAGAGCTCAAACTGAAACTAGAGCTGAAACTAGAGCTGAAACTAGAGCTGAAACTAGAGCTGAAACTAGAGCTGAGACTAGAGCTGAGAACCATCCTGTTACTTCTCCCCATATTAGA
GCTAGTGATACTTCTGTTATTTCCTCCCACACTAGAACTGTGAATCACCAATCTCCCCATACTAGAGTCAGAGATAGTTCTGTTAATTACCCACAGACTAGAGCTGAAGAATTTTCTACTCCC
CATTTTAATCCTGAAAATCGTCCGTCCGGAACTGGAGATCATGACACTGGAATATACCCTGTTCGTCGGAGACGAGCCGTAACAAACCTAGCCCAATATTATAATCGACGTTAA

M-*orf* (26 sequences)

>h_EchMenM_Morf
ATGTGAGGCCAGAATGAGCTCCATAGGATATCGGATTTTGTGTTGGAGTATTCTGACTTTGTTTGTTTTCTTTTGTTAGTTATCATTAGGACTTGGAGTGTTTTTGCTCGTGCTGCTAAGCTT
ATTGGAGTTTCTAAAGCTTGGGAGACACTAAAACGATATTGGTTCTCCCTTTTTGTTTTGTGGGTTGGTTTTGAGGTTTTTCTTTATATTACTTGGACTTGTGTCTATAAGTTGTTTACATAC
TTGTCTGATCCTATTCGTGGATTATTCTTCTTGTCTATGGTTTTTGGGTTGCTTACTTTTTGGGCTTTGTGGTTGTTTTTTGATGGTATTAGGACGGGAGGTTATTTTAGGTTAGGGGAAGAG
GTTTCTGCGGGTAGTTCTACCGCAAAGAGGGGGAAAGGTAAAGGTAAGGTTTTGAAGGGGGGTGTGGTGGAGGGTGATTTGGCTTTAGGGGGCGAGGTGACAGAAGGTTTGAGGGATGATGCT
GGTGATGATAAAACTAAGAAGGTAAATAAAGAAGGTACAGATAGCCCTAAGAAAGTGAAAAAGGAGACTAAGGATAAGCCTAAAAAGGTAAAGAAGGAGGTTACAGGTGAACCTGAAGAGGTA
GAGAAAAAGGTTATAAATAAAGCTAAGAAGGTAAAGAAGGAAACTGCGGATGAGCCTGAAAAGGTGAAAAAAGAGGCCGGTGATAAGCCTAAGAAGGTAAAGAAAGAGCCTGCGGATAAACCC
AAGAAGGTAAAGAAGAGGGCTACAGATGAGCCTGAAAAAGTGAGGAAAGAGGCCGTAAGTAAGCCTAAAAAGGCAAAGATAGAGTCTGTGGGTAAGCCTAAGAAGGTAAAAAAAGAGGCTATA
AATAAGCCTGAAAAAGTGAAAGTAAGGGGGGGGGCCACCGATAAGCTTGAGAGTGATGGTAGGGATAAGGGTAATAAGTTGCAATAG
>h_WesCarM_Morf
ATGATAGGGATGCCTCTATTCGATACTTTGGAACTAGTGCTTGTGAGGTACCCCAATGTTGTTTGTTTTTTGGTGTTAATGTTTTCAAGTACTTTTGGGATTTGTGCTATTTCTGTTAATATG
GTTGGTTACCACAAGATGTGGAAGTTTTTCAAGTCTTCTTGGTTTTATTGTTTGTGAATATGGGCTATTTTTGAGGCGTTTGTTTATGGTATTTGAGTTTGTTTGTTTATTGTTTTTGATAGG
GTGTCTGGTCCTGTTCTTGGTTTTATGTTCTTTACTGTGGTTTTTAGGTTGGCTGTTGTGCGGTTATTGAAGTTATATTTTGAGATAGTCACTGAGGTGGATGATGTTAGGCCTAGTGGTGGT
GATGGGTCTGTGGTGGTAGATAAGGAGACTGAGGTAGACGAATTTAAGGGGGCTGTTAAGGGTAAGACTAAAAAGGGGGATGTAAGTAAAGCTAATAAGGGGGTTAAGGACAAAGTTAAGAAG
GTTGAGAAACCTGGTGTAAGTAAGCCAAAGAAGGTAGATAGAAGGAAAGCTAAGAAAGTTGACAGGAGTAAGGTTAAAGGGGTGGTTAGGAGGAAAGCTAAGAAAGTTGATAGAAGTAAGGCT
AAAGGGGTGGTTAGAAGTAAAGCTAAAAAAGTTGATAGAAGTAAGGTTAAGAAGGTGGTTAAGAGGGGAGCTAAGCAGGCTAGGAAAGTTGGTGTAGGGCTTGGTAAGGGGAAGGGGAAAGGC
AAAAAGTAA
>m_CumMonM_Morf1
ATGAAAGCAACCCTGTGTAAAGTCATTGAGTTTGTATTGGATAATGGTTGGCTTTGTTTGTTTTATTTTGTTTTGTTTATGGCTTGTAGGAATGTGCTGTGACGTGTGTACAAGGTCCGTAAG
GGTTTATACAAGAAAGTAAAGCGTGTTATTGGAGTTATTTGGAAGCCGACTGCGGTTTCTGTTAAAATTAAGAAGGACAAAGTAGAGAAGCCTAAGGTTATGGAGAAAGCGAAGAAGGGGAAA
AAGGCTGCGGTGTCAGGTAAAGTGGGTAAGAAGTCTGGTGGTTAG
>m_MarMarM_Morf1
ATGAAGGAGCGGCTATGCCAAATCATTGCTTTTGTGGCGGACAATAGGTGGCTTTGTGTGTTTTACTTTATTTTATTTATGGCTTGTATAAACGTAGTGAGGCGCTTGTACAAAGTTCGTAAA
CTTCTGTTTAAGAAGATAAAGCGTATTATTGGGGTTTTGTGGAAGCCGACTGCGCCTGTCATTAAAATTAAGAAAGAGAAAGTAGATAAGGTTATTGCGGTAGATAAAGTTGTAAAAAAGGGC
GGGAAAGCCAAGGTGGCTGGTAAAGTGGGTAAGAAGTCTAATTAG
>m_PseMrcM_Morf1
GTGAAAGCAACCCTATGCAAAATCATTGAATTCATATTGGACAATAGGTGGCTTTGTTTGTTTTATTTTATTCTATTTATGGCTTGCATGAATGTGCTGTGACGTGTGCACAAGATTCGCAAA
GTTTTGTATAAGAAGGTTAAGCGTGTAGTTATGGTTCTTTGGAAGCCGGTTGTGCCCCCTGTTAAGGGCAAAAAAGAAAAGGCGGATAAGGTTATCGTAGTAGAAAAGATTGTAAAGAAAGGC
AAAAAGGGTGGGGTTAGCGGTAAAGTGGGTAAGAAGTCTACCAGTTAG

```
>m_CumMonM_Morf2
GTGGTGTGCTTAAGAAGGGTAGATTGTGGGGTTGAGGTCCCGCTTGTGCTAGATGGTGCCCTTTATTTGAAAGCTCTTTTTGGGGCAATTCGTGAGTTCTTTCACGAAAACAGCTTATTTTTA
CTTATTATTGCAGTTTTGGTTTGTATTTGTGTGAGTGCGTTTATGTGAGTTTATGAGCGGCGGAAGGTTTTTGTTAAGAAAGTTAAGCGAGGGCTTAAGGGATTGTGGAAGAGCATTAAGGTT
GACTTTAGAGTGGGGGGGAGTGGTTGGGTGCGGTTAGGGGCTATGTCAATAAAAGTGTTGAAGTGATTTAGTCGGTTTTGTGTTCGTGTGCTTTGGCTTGTCACCCTATTTGTTCTTCTTTCG
GTTTTTTTAGGGTAA
>m_MarMarM_Morf2
ATTCCCTATTTGAGGGCTATCTCCGAGTTTCTATCAAAGAATGGATGGTTTTGTGTGGCTTATTTAGCATTATTCTGGGTTTGTGTAGATGCATTGTGGCGGGCGTATAAGGTGCGTAAAATT
TTGCTTAAAAAAATTAAGCGCCTGAGTAAGGCTTGGTGAAAAACCATTACATTTGGGGTAGCGAAGATAAAGAATAGAGTAAGGGTTTCATTGTGATTGGGGTTAGTGGTGGCAATAAGCGGG
CGCTCAAAGGTAGTAGCGGTAAAAGCAGTTAAATGGCTGAAGAAATTTTGCGTTAATGTTTGCTGGGTTATTCTTTTTATAGTTTTACTCACGCTTCTTTTTAGCTAG
>m_PseMrcM_Morf2
ATTGTAGAGGTTATGGGTCTCTATGTGTTACTGTATGGTATCCCTTATTTGAGAGATGTTATTGATGCCATTGTAGAGTTTTTGTCGAAGAGCGGTTGATTTTGCGTAGTTTACTTAGCCCTG
TTTAGGGTTTGTGTCAGTGTGTTTTTGTGGGTGTGGGGGAAATGGAAGATTTTTGTAAAGAAAATTAAGCGTATTAGTAAGGCTTTTTGGAAGTTTGTTGTATTCTGGAAAGTTAAACCCAAG
AAAGGTGGGATGGGCAATAAAATTAATGTAAGTAATGGCGGGTGGTGAAGTAGAGCAATGAAGGCAATAAGACGGTTAAAGAATTTGTGTGTGAAGGTTTGTTGGGCTATTTTTTTGTTTATT
CTTCTCATACTTCTTTTGGGGTAA
>u_AcuTorM_Morf
TTGCAAATAACCAGTGATTTCATTAAGTGAGTGAAACATTGCTATGGGCTTTCTCCTTTGCTTACCTTAACCTTTTTTGTTGTTGTTGGGTTAATGGTTTTTGGTCTGTTCCGTGGGATTTTT
TTGTATTGATCTAAAATTTATAATGGTTTTATGCTTGTAGTCATAGGAAGCAGGTGATTAACTGAGAAACTTGCCGGTGAGAGTAAAGACGAAAAGGGTGCTGTGAGTGATCAAAACTCCTTA
AGGGAGAAGAAAGTTAATGGCGTTGTTGAGGGTCTTGTTGAAGGTGTTGTTGGTGAGGTTGTTGGTGGGGTTGAGATTGTGGCTAATAAAAGAGATTCTAAAAAAGTTATGGATGTAGATAGT
TTGAAAGATATTATTAAGGAGGCTATTAAGGAAGCTGTTAAAGAGGCTATGAAAGATCTTGTTGTTAAAGAAGCTAAAAAAAAGAAAGAAAAGGTCGTTAGAGGGGATGGAGTTACACCAAAG
AAAAAGAACAAAAAGGTTTTGGCCGAAGAAGCGAGAGGGGTAGGTGTTGGGTTAAGTGGCCAAGAGGTGGTTCCAGTGAAGAAAAAGAAGTCTTCTAAGAAGAAAGAGTTAGTGACTAAGGAA
ACTGTTTCTGAATAG
>u_AnoAnaM_Morf
ATAATGGATCTACTCAATGACCTAATCACTTTTGTAAAGTGAATCAAGAATTGTTTTGCTTTGTCCCCATATGTTACTTTAGCCATGCTTTTTGTATTTATGATAGTTATTGTAGGAATTCTT
CGTGGTATTCGTTTGTACGGGTATGATTTGTATAAAAAATTTATGCTTTTGATAGTAGGAAGGAGATGGTTTACTGAAAAGTCTGGTGAAGCAGTTAAAGACGAGAAAAGTATTAGAGATGGT
TTAAACCCTTCTGAGGGTCAGAAGGTTAGTGGTGTTGATGGGCCTAAGGGGGAGGGGGGCTCGATTAAAGTTGAAGATATTAGTAGTTTGAAGAATATAATTAGTGAAGCTGTTAAGGAAGCT
ATTAAGGAGGCAATAAAGGATCTTGTTGTTAAAGAAGCTAAAAAGAAGAAGGAAAAAGTTGTAAGTGGGGATGGAACTAGACCAAAGAAGAAGAGTAAGAAGTCTTTAGAGGTAGGGGAAGCC
GTAGGAGATACTGTTGTGGTTGTGTCTTCTAAAAAGAAAAGTAGCCCTAAGAAAAAAGAATTAGTGGTTAAGGAAGAACCTTCTAAAGAAAATTAA
>u_LamLeaM_Morf
ATCGTGAAGGATATGTATAGGGCTTCCCCGTGTGGTACGGTTTTTGTTTTTTTCTTGTTTTTTTTGTATATATTTGTGGCGATTTACTCTATGTGGGTTGGTCCTGAGGGGCATAAAAAAGTA
GTTGATCGAACAAAGAAAGTGGTATGGGGTAGTGGTAAGACGATAAAAGAAACAAGCGCTAAAGAAAAGAAGGTAACTAAGAGTAAGAAGAAAAAACAAGAAGGTGTTGGTGTGGAGGAGGGG
CCTCTGCCTGGTGTTGTTGCGGCCACAGCCAAGAGGAAAAAGGTGAGTAAGAAACCTAAGGATGGGGAGTAG
>u_LanLanM_Morf
ATGGTTATGTTGCATGATCTAATTAAATGGGTGAAACATAGGTATAGACTTTCTCCGGTTCTTACCTTTACTTTTTTTGTTGTCCTTGGTCTGATGGTTTTTGGTTTGTTTCGTGGACTTTTC
TTGTATTGATCTAAAGTTTACAGGCAAATTATGTTTTTACTGATAGGAAGTAGGTGGTTGACTGAAAAGCTTGCGGATAGAAGTAAAGGTGAGAAGAGTGTTGGTGATAATCAAAATTCTTTG
AGGGATAAAAAAACTGATGGTGTTGCTGGTGAAGTTGTTAGTGGAGTTGAAGTCGGTAATAAAAGCTTTGAAAAGTCTATAGATATTGGTGGTTTGAGTGATGTTCTTAAGGAAGCTATTAAA
GAGGTTGTTAAAGAAGCTGTTAAGGAAGCTATGAAAGATCTTGTTGTTAAGGAGGCTAAGAAGAAAAAAGAAAAGGTTAGTAATGGTGATGGGGTTGTACCAAAGAAGAAGAGTAAGAAAGCT
TTGGTTGAACAGGCTGGTAGAGTAAGTACTGAGTTAGGTAATCAAGAGATGGTTTCTGAGAAGAAAAAAAAGTCACCAAAAAAAAAAGGAGTTGGTGAGTAAGGAGACTGACCCCAGAAAATAA
>u_PotAlaM_Morf
ATGCGTGGGGTCTGAGAACTGGTTCGTTGAGTTGAGTACGGGCTTAGTAATTACCCCATTATAACTATAGGTATGTTGGTTTATACTGGCGCGGTAATTTTTGGATTTATTCGTGGTCTTATT
CATTTAGAGGAATACTTTAAGGAGAAGCAAGAAAAGAAAGAAAAAATAGAAAAAATATTAAAAGAGAAGTTTGAGGCTAAAAAAAAGACAAAAAAAATAAATATTGTTAGTAAAAAAGCTGGG
GGGGTGGCTAAAATAGAGGGAGTTAAGAAGCTTAATAAAAAGACTAAGAAAATTACTGTTGAAGAAAATGGAGTGGATGGGGGTGCTAAAAATGTTGTTAAAAAGAAAAAGACTATTGTTGTT
AAAGATGTGGTAGGTGGGGAGACCAATAGTACTAAGAAAAAAAAGATTATTACTTTGAAAGGTAAGGTAGGTGGGGATACCAAGAATATTGAAAATAATAAGGTTGTTGTTAAAGATGCGGTA
AGGGGTGAGGCTAAAAATACCGTTAAGAAGAAAGTTGTTGTTAACAAGAAAGCTAATAAAAAGAAGAGTAAGGTAGTGGTTAAGAAAAAGCCTAAGGGGGATAATGGAAGGGCTGAGTAG
>u_PotLitM_Morf
ATGAGACGTGCCCCAAATGCATTCATCGAAATCGTGAAAGAGATGTATAGGGCTTCTCCATGCGGAACAATTTTTGTTTTTTCTTTGTTTTTTTTATACATGTTTATGGCACTTTATGCTATT
TGTGTTGGCTATAAGGTGCATGAAAAAGTTATAGGTTGATTAAAGAAGACGGTGTGGGGTAGTGGCGAAACAGTGAAGGGGGGCTAACGTTAAGGTTAAAAAAAAGGCTAAAGCTAAAAAGAAA
AAAGAAGAGGGGGTAAGTGCGGTAGGAGAGGGGGGAGCATTGTCAGAGGTTGGTACGGCTTCCGCTAAAAAGAAAGTGGTTAGGGAGCCTAAGGATAGGCAGCAGTAA
>u_ProJapM_Morf
ATGAAACGTACCCTGGACATGTTTGTTGAAATTGTAGAAGAGATATACACGGCTTCTCCGTGCGGTACGGTTTTTGTTTTTTTTTTGTTTTTTTTGTATATATTTGTGACAATTTACTCTTTG
TGGGTTGGTCCTGAAGGGCATAAGAAAGTTGTCGATAAAACGAAAAAGGTAGTGTGGGGTAGTGGTAAGGCAATGAAGGAGGCGAGTGTTAAGGGGAGGAAGAGAGTTAAAAGTGCAAAGAAG
AAAAAGCAGGAGGGTGCCGATGTAGGGGGGGAGGGGTTGTTGTCAGATGCTGCGGTTCCGGTCAAAAAGAAAAAGGTGACTAAGAAACTTAAAGAGCAAGGAAGTAAGTAA
>u_PygGraM_Morf
TTGCTACATGACGACTTACACCTTCTTGTTAAGTGGTTAAAACACTGTTTCTCTTTGTCACCTTATGTGACTTTAACTATGATTTTTGTATTTGGGTTGATTATTTTTGGGTTTTTTCGGGGG
ATTTATTTATACTGGCACGAAATTTACAAGAAGTTTATACTTTTAGTAGCTGGAAAAAGATGATTTATTGAAAAGTCTGGTGAGGGTAGAAAAGATGATAAAAATATCAAGAATAATTCTGAT
ACTTTAGAGGGCTCTAAAGTTAAGGATACTACTAGTAATGTTGAGGTCTCTAAGGATTTGGAGGTTAAAGATACCGATAGTAACTTTGGGGGTTTAAAGGTTACAGATAGGGTCAATGATGTT
GGTATTTTTGGCAGGCCTAAAGCTATGGGTGTTGCTGGTGGTGTTGCAGTCTCTAAAGAAAAAGCTGGTTCTCCGGAAGAAGAAAAGAAGAGTGATTTAATAGATACTATTAAAAAAGCTGTA
AAAGAGGCTCTAGAGGAAGCTATGAAAGATTTTGTTGTTAAAGAGGCTAAAAAGAAGAAGCAAAAAGTTGCAGGGGAAAACCCAACACCAAAGAAAAAAAGTAAAAAATCAGAAGGAGTGGAA
GTAGATTCTGTTAAGGTTGTAACCCCAAAAAAGAAGAAAACCTCTAAGAAAAGAGAGTTGGTGATTACGAAAGATCCTATTAAAGAATAA
>u_QuaQuaM_Morf
GTGAAGGAGGTTCTATCAATTTCTTGGTTTATTTTTTTTGTTATGGTTTTTTTACTTGGGTTACTGTTTGTTGGGTTTATTAAAGGGATTTTGGTATTTTGGGAGATTTTTGAAATAAAAAGA
TCGGATGTTGGAGGAAAGGTTAAGGGGGTTTTGAAGAGTAAAAAGGAAAATAAGGCTAAGGTACGTGGTAGGGACTTTGGTAGTGATGGTGTGGTTAGTAGTAGCTCTCCAAGGAAGAAATCG
GTTAAGAAGAAACCAAGGAAGAAAAGCGGGGCGGTGCTTAAGGATTTAAAAAAAGATGATGATGGTTCTTAG
>u_SchspM_Morf
ATAAGAACTTTTAGTAATACACTAAAATGAGTCAAGCATTGTTATATGCTTTCGCCTGTGCTTACTTTTGTTTTGTTTTTTGTTGCTGGACTTATAATTTTTGGTTTGTTTCGTGGTATTTTT
TTATACTGGTCTAAGATTTACACTTGAGTTATGCTTTTGTTAATGGGAAGCAGATGGTTTTCTGATAAGCTAGCTGATGGTGGTAAAGATGAGAAAAGTGTTGATGGTCAAGGTTCTGTGGAT
GGAAAGAAAGTTGACGGTGTTGTTGGTGAGGTTGGAGTTGTAGTTAGTAAGGAGGATTCTGGTAAGTCTATGGATGTTAGTGGTTTAGTGGATGTTTTGAAAGAAGCTGTTAAGGAAGCTATT
AAAGAAGCGATGAAAGACCTTGTGGTTAAAGAGGCTAAAAAGAAGAAGGAAAAAGTTACTAGCGGAGATGTAGCTGTTGTACCTAAGAAAAAAAATAAGAAGGCTTTAACAAGTGAAGTAGGG
GGAGTGACTGTTGGGTTAGATAATCAGGAAGTAGTTGTTGCAAAGAAAAAAAAAATCTTCATCTACTAAGAAGAAGGAGTTGGTGCCTAGGAGAGTCGATGTTGAAAAATAA
>u_SinWooM_Morfa
TTGAAGAGATGTAGGGTTGTCAACTCTAAGAAGTTAGTTCTTAACACCTCTTTAATGCTGAAAAACATAACAGAATTTACCCAGTGGATTAAGCTTTGTTTCTGCTTGTCTCCCTATGTTACT
ATTACTATACTATCTGTGCTTTTTCTAATTCTTTTTGGACTTGCCCGTGTAGTTTATCTTTATGGGTTTAGTGTTTTTAAGATGTTAATCACTTTTATAATAGGTGGAAGGTGATTTACTGAT
AAGTCCAATGAGATTATTAAAGATGAGAAGGTTGTAAGTGATGGTCAGGGTTCTTTGGATAGGCGCAAAGTTTTGGGTAATTCTAATAGTGATTCTAATAGTGATTCTAATTATGGTAATAAA
CCTGGTGGTGATGTTCCTTTAGAGGAACAGGGTTCTTTAAAAGTTGACGATTTTGGTATATTAAAGGATTTAATTAAGGAGTGAGTCAAAGAAGGTATTAAGGAAGCCTTAAAAGATTTTGTT
GTAAAAGAAGCTAAAAAGAAAAAGGAAAAAGTCATTAAGACAGAAGGAACTTCACCAAAGAAAGGTAAAAAGGCCTTAACTGTGGGGGAGAGAGAGGCAGTCTCACCTAAGAAGAAGAAAAAG
AATTCTAGTAAGGGAGAATTGGTGAGTAAGGATGTAATTATTAAAGAATAA
>u_SinWooM_Morfb
ATTTGTTTTTTTGAGTTTAAACTTTTTAAGAAGTTTGTGTCTTTTATGACAGAAGGAAATTGGTTTTCTGAGAAGTCTAGTGAGATTATTAATAATGAGACTATTAAAAATGAGAAAGTTCCT
AGTGATAATAAAAGTCCTTTGGGGAGGCAGGAAATTGTGAATGATTCTAGTAGTGTTAGTGATACTAGTTCTTTAAAAGACTCTGTTCCTTTAAAGACTGGTGATATTGGTAATTTAAAAGAC
ATAATTAAAGAGTCTATTAATGAAGGTATTAAGGAGGCCTTTAAGGATCTTGTTGTAAAAGAAACTAAGAAAAAAAAGGAAAAAGTCGTTAAGACAGAAGGAACTTCCCCTACTTCCCCTAAG
AAAAGTAAGAAAACTTCTACTAAGGGTGAAGTTACAGCATTTATTTCTGGGGTGGTTTTAGAGGATAGTGATCCTATTAAAAAAAAGAGTTCTACTAAAAAAAAAAGAGTTGACTACTAAGGAA
GAGATTCCTAAGAGGTAA
>u_SolCarM_Morf
ATAAATGCCTTAAAGACATTTGCTGAGATTGTAAAGGAGATGTATAGTGCTTCTCCGTGTGGTACGTTTGCTTTTTTTTTCCTTTTTTTTTTGTATATTTCTGCAATGATTTATCTTATTTAT
ACGGGTCCGGGAGTTCATAAAAAGTTGTTTGGGAAAATTAAGAAACTGATTTGGGGTAGGGGTAAGCTTATGGGGGGGAAGGTTGATGCTAAGGGTAAAAAGGTTAAGGCACCGCGTAAAAGC
AAGAAGGATAAGGAGAATGAAGTGGAATCTTTGGCAGCTGTTTCAGAGATTGTACCTAAGAAGGCAAAGAAGGTAAGTAAGAAATCCAAGGATGATTCTGCGACTAAGAAGCCTGTGGATGAA
CCTGTGATTAAGGGGCCTGAGGATAAACCTGTGATTCAGAGTTCTGAGGGTGAGGTTAAAAGTTAA
>u_UniCraM_Morf
ATGCATGATATCGTAAAGTGGGTTAAACACTGCTTTAGAGTCTCACCTGTTGTCTCAGCAATTGTTTTTTTTATTTTATTTTTTGTGTTTGGGTTAATGATTTTTGGTTTATGGCGTGCCATT
GATTTATATTGATATAGAATTTATAGTGCGATTATGGTTGTGTTGATAGGGAGTAGGTGGTTTGATGATAAGTCTGTTGATAGTAATAAGGGTGAAAAAGTGTTTGCTGGTAATAGTCCAGTT
TCTTTAGATGAAAAAAAAGTTGGTGATGTTGTTAGTGGGGTTGAGACTTTGGTTGTCGATAAAGAAGATTCTAAAAAGGGTATGGATGTTAATGATTTAACGGAGATTATTAAAAAAGCTGTT
AAAGAGGGTATTAAAGAAGCTATGAAGGATTTTGTTATTAAAGAGGCTAAGAAAAAAAAAGATACAGTAGTTAGTGGAGATTCGACTACACCAAAAAAAAAGAGTAAGAAGGTTTTGGCAAAA
GAAATGGGTGAAATGGTTAGTGAAACAAATAATCAAGAGGTAACTCCAACAAAAAAGAAGAAATCTAAAAAGAGGGAGTTAGTGAATAAGGAAGTTGGTCCAGAGAAATAA
```

>u_UniDelM_Morf
ATGAATATCACCCACGACCTAATTAAATGGGTCAAACATTGCTTCGGGCTCTCTCCTGTTGTTGCTTTTATTATTTTTTTTATTTCTTTTTTGTACTTAGGTTAATGATTTTTGGTTTGTTT
CGTGCTGTTTCTTTGTATTGATCTAGAATTTATAATTCGACTATGATAGTGATAATGGGAAGTAGGTGATTTGTTGATAAGTCTGCTGGTGATAGTAAGGACGAAAAAGTTGTTGTTAACGGT
TCGATTTCTCTTGATAAAAACAATGTTGGTGAGGTTGGGACTGTCGGTCTTTGTGAGCAAGATGACTCTAAGAAATTTGTCGATATTAGTAGCTTAAAAGATATTCTTAAGGATATTCTTAAA
GAGATTGTTAAAGAGGCTGTTAAAGAGGCCATGAAAAATGCTATGGTTAAAGAGGCTAAAAAAAAGAAGGATGGGGCTGTTAGAGCTGATTTAACGGCAGTAAAAAAGAAAAGTAAAAAGGTC
CTGGCAAAGGAAATGGGTGATACAATTAGTGAAGTGGATAATCAAGGGACAACTCCGACAAAAAAGAAGAAATCTAAAAAAGTGGAGTTAGTGGATAAGGAAGTTGATTTAAATAAGTAA
>u_UniTumM_Morf
ATGGTCATGCTGCACGATTTTATTAAGTGGGTTAAGTATTGTTGCGAGCTTTCTTTTTTTGTTACATTACTCTTGTTTTTTATTGTAAGATTAGTGATTTTTGGCTTTTCCGTGGTATTTAT
TTGTATTGGTCTAAGGTTTATAGTGGGATCATGTTTGTAATAATAGGGAGTAGATGATTTACTGATAAGTCTTTAAGTAATAGGGGAGACGTAAAAAGTGTTAATGATGGTCAGAGTAGAGGT
AATAAGGGTGATGTTAGTGTTAGTGATAGTCATAATTCTTTAGGTGAAAGGAAGGTTGGTGGCGTTGTTGGTAAGGAGGATTTTGGAAAGGTCATAGATGTCGGTAGTTTGAGTGATGTTTTA
AAGGAAGCCGTTAAGGAGGCTATTAAGGAGGCTATGAAAGATCTTGTGGTTAAAGAAGCTAAGAAGAAGAAGGAGAAAGTTGTTGGTGGGGATTCAACATTACCAAAAAAAAGAAGTAAAAAA
GTTTTAGCGGGTGAGACGAGTGGGGTGGTTGTTGAGTTGGGTAATCAAGAGGTAGCTCCTGTAAAGAAGAAAAAGTCTAAGAAGAGTGAACCAGTGATTAAGGAAGTTTTCCCTGATAAGTAA
>u_UttPenM_Morf
ATAGAAAACTTTAGCGTGTTCTTCAAATGAGTTAAGGATTGTGTTATGGTGTCACCTTATGTGACTTTTGTTATGTTTATAGTAGTTATACTATTAGTTCTTGGATTTGTTCGTAATGTTTAT
TTATACTGATATGAAGTTTATAAGGCATTTATAACCTTGATAGTAGGGAAAAGGTGATTTACGGATGACCCTAGTGTAACTACTAAAGAGACTGACACTACTAATCGTAGTTTTGATCCTTCT
GGTGAACCTAAAGTTGTTAGTAATGTTAAAATTGTTGAAGACTCTAAAGTTGTGGATATTACTGGTGGAGTTAGTACTTCTGTAGATTCTGGAATTAAGGATAATAAAAGTGGTAGTGATGCT
CCTAAGGAAGCAGTTGGTTTACCTAAGGTTGAGAATAAAAATGATTTGAAAAGTACTATTAAAGAAGCTGTTAAAGAGGCTTTGGAGGAGCTTGTGAGAGAGTATGGTATTAAGGGAATTAAA
AAGAAGAAGCCAGTTGGGGAAACTGCTGCACCAAAAAAGAAAAGTAAGAAGTCTTTAGAAGGAGAAGTAAGAGGAACTGATCCTGCTAAGGTTACAGTTCCTAAAAAGAAGAAGACTCCTAAA
AAGGGGGAGTTGGTGATTACGAAAGATCCTGTTGGTAGCTAA
>u_VenEllM_Morf
ATCTTACGGCTGATCTCAGACCTAGTTAGATGGCTTGGGTTTTGCCTAGAAAACTACCCAATTCTTACTTTGTTTATGTTGTTCTTTACCGTGCTTATATTTTGGGGGTTTGTTCGCGGTATT
GTGACTTTAACTGAGGTCTTTGAGGAGCAACAAGAGAAGGAAGTTGCGTTGGGTAGGTTAAATAAAGATAAACTTGAGTTTGAGAAAAATATGGGGAACCTAAAAATAATAGAAATTGAACTT
AACAAAAAGATAAAGGCGTTTGAGCTTGATAAAAAAGTGGATCGTTTGAAAAAAGAAGAATTTGGACTTATTAAAAAGGTAGATGCTTTAAAAAAGGAGGAATTTAAGTTTCAAGGAAAGTTG
GAAGAGTTAAAGGCAGAGGTATTTGAGCTTCGTAAGAAGGTGGATAAGTTGAAGGAGGAGGAGTCTATAATCGAGGAAAAGGTAGATATGATAAAAATAGAGTGGCTTAGACTTGATGTAAAG
ATAAACTCACTAAAAAAAGAGGAATATGAATCAAAAAAGGCTGACAAAGAAATTGAGGGAGATGACATCAAAGAAAAAGAGAAAGTTTTTGATATTGTTGATGATGAGGTTGGGGTGGAAGCC
AAGAATATTGATAAGAAAAAAAAACTTGCTAAAAAAAGTTGGGGGGGTTACTAAGAATAGTGACTAA

Anodontites trapesialis **ORFans** (2 sequences)

>y_AtraUR219
ATTGAATTTTGTATTAGGGTTATGGGTACCAGTTGTGGTTTGTTTTTATTATTCTTTTTATGTTTAATTTTATTTAGTAGAGATAGGTATAAATATGGATTTTTTAATTTAGTTAGGGAGAAG
GTGGTCAGGAGGATTTATAATCTTTTGAGAGTTGTTTTACCTCCAAGGCCTAAAAAGGGTAAGCCTAAGATTAAAAAGTAA
>y_AtraUR2218
ATAATTGTTGGTGTTTTATCTTGGTATGTTTGTTTATTCTTTGTGTGTTTGAGTTTATTTGTGAGGTAG
